# The Molecular Basis of Toxins’ Interactions with Intracellular Signaling via Discrete Portals

**DOI:** 10.3390/toxins9030107

**Published:** 2017-03-16

**Authors:** Adi Lahiani, Ephraim Yavin, Philip Lazarovici

**Affiliations:** 1 School of Pharmacy Institute for Drug Research, Faculty of Medicine, The Hebrew University of Jerusalem, P.O.Box 12065, Jerusalem 91120, Israel; adi.lahiani@mail.huji.ac.il; 2 Department of Neurobiology, The Weizmann Institute of Science, Rehovot 76100, Israel; ephraim.yavin@googlemail.com

**Keywords:** ion channels, lethal dose, neurotoxicity, phospholipids, plasma membrane, protein kinases, receptors, second messengers, signal transduction portals, toxins

## Abstract

An understanding of the molecular mechanisms by which microbial, plant or animal-secreted toxins exert their action provides the most important element for assessment of human health risks and opens new insights into therapies addressing a plethora of pathologies, ranging from neurological disorders to cancer, using toxinomimetic agents. Recently, molecular and cellular biology dissecting tools have provided a wealth of information on the action of these diverse toxins, yet, an integrated framework to explain their selective toxicity is still lacking. In this review, specific examples of different toxins are emphasized to illustrate the fundamental mechanisms of toxicity at different biochemical, molecular and cellular- levels with particular consideration for the nervous system. The target of primary action has been highlighted and operationally classified into 13 sub-categories. Selected examples of toxins were assigned to each target category, denominated as *portal*, and the modulation of the different *portal’s* signaling was featured. The first portal encompasses the plasma membrane lipid domains, which give rise to pores when challenged for example with pardaxin, a fish toxin, or is subject to degradation when enzymes of lipid metabolism such as phospholipases A_2_ (PLA_2_) or phospholipase C (PLC) act upon it. Several major portals consist of ion channels, pumps, transporters and ligand gated ionotropic receptors which many toxins act on, disturbing the intracellular ion homeostasis. Another group of portals consists of G-protein-coupled and tyrosine kinase receptors that, upon interaction with discrete toxins, alter second messengers towards pathological levels. Lastly, subcellular organelles such as mitochondria, nucleus, protein- and RNA-synthesis machineries, cytoskeletal networks and exocytic vesicles are also portals targeted and deregulated by other diverse group of toxins. A fundamental concept can be drawn from these seemingly different toxins with respect to the site of action and the secondary messengers and signaling cascades they trigger in the host. While the interaction with the initial portal is largely determined by the chemical nature of the toxin, once inside the cell, several ubiquitous second messengers and protein kinases/ phosphatases pathways are impaired, to attain toxicity. Therefore, toxins represent one of the most promising natural molecules for developing novel therapeutics that selectively target the major cellular portals involved in human physiology and diseases.

## 1. Introduction

**Figure 1:**
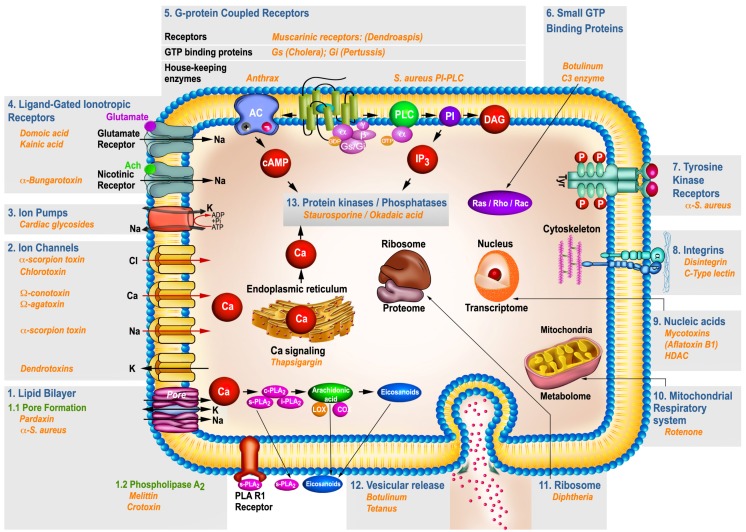
Overview of the signal transduction portals (**blue**) modulated by bacterial, plant and animal toxins (**orange**). The portals presented are numbered from 1–13 (starting from bottom left side) indicating the paragraphs in Section 2 where they are discussed. The left part of the figure highlights ionic channels and ionotropic receptors, which regulate intracellular ion concentration and may lead to Ca^2+^ overload, as well as to a separation of plasma membrane lipid domains and pore formation **(green**); the top part of the figure illustrates the G-Protein Coupled Receptors (GPCR) systems, generating second messengers such as cAMP and IP_3_. The right part of the figure describes tyrosine kinase and integrin receptors, and indicates the targeting of nucleic acid and the mitochondrial respiratory system; the bottom part presents ribosomes and vesicular release. Protein kinases and phosphatases activated by second messenger, are indicated inside the cell.

Generation of toxins by a wide variety of organisms ranging from single cells (i.e., bacteria, fungi) to complex organisms of the plant and animal kingdoms (i.e., scorpions, reptiles) is a well-known phenomenon in cell biology, enabling certain organisms to respond to their environment in a defensive or offensive manner. The number of toxins isolated and identified has constantly increased over the years and better insights into their molecular structure and mechanism of action have been achieved. A vast majority of these toxins act by either modulating essential signaling cascades in the eukaryotic cell [1] or by direct interference with the ionic equilibrium maintained by the cell membrane barrier. With respect to the latter, some toxins act by direct insertion into the lipid bilayer to form pores while others act on ion pumps or ion-gated channels responsible for maintaining the ion gradients (Figure 1). The molecular mechanism underlying the interference of a toxin with a signaling cascade is directed at a specific target on the cell membrane where regular signals or second messengers initiate a normal physiological response. Upon interaction with a toxin, the subsequent biochemical chains of events are perturbed, and in most cases, a pathological response is evident. These targets are rather distinct at the membrane level and include diverse groups such as ligand-gated ionotropic receptors, G protein-coupled receptors, tyrosine kinase receptors, integrin receptors and certain lipid species present in the bilayer plasma membrane of the cell. Some targets are localized inside the host cell, and consist of functional organelles, which react specifically to some but not all toxins. Two major mechanisms of changes in the target protein receptor at the plasma membrane level are distinguishable. One mechanism consists of conformational changes such as the binding of the acetylcholine molecule to the alpha subunit of the nicotinic receptor followed by allosteric transition and opening of the channel for ion–induced cell depolarization [2]. A second mechanism of protein modulation involves translocation, as exemplified by the stimulation of the skeletal muscle cell by insulin, which causes contraction-induced glucose transporter translocation from the intracellular vesicles to the outer plasma membrane domain to enable glucose uptake [3]. The changes in membrane lipids brought about by toxins containing hydrolytic enzyme activity such as phospholipase A_2_ (PLA_2_) or phospholipase C (PLC) are notorious since they may generate lytic products and restructure the lipid bilayer [3,4,5]. Other toxins act as pores to interfere and collapse the intracellular ionic equilibrium. 

Once the toxin-host cell interaction is initiated, vital intracellular processes such as energy metabolism, posttranslational modifications, cytoskeleton stability, gene expression, motility, secretion, cell division and other specific functions, may be compromised. Under physiological conditions, interaction of cell targets with their natural ligands results in controlled changes of intracellular levels of second messengers such as cAMP, Ca^2+^, inositol 1,4,5-trisphosphate (IP_3_), or 1, 2-diacylglycerol (DAG). Concomitantly, protein kinases activated by these second messengers, phosphorylate and activate many cellular substrate molecules amplifying the signals. When the priming of this process is compromised by a non-physiological stimulus such as a toxin, the entire cascade is perturbed and adverse cellular responses including cell death may occur [1]. The toxin can act as an agonist or antagonist, enhancing or inhibiting certain enzymatic activities, thus impairing intracellular signaling cascades resulting in pathological outcome.

With that in mind, the present review aims to revisit and put forward a conceptual bridge between a group of different toxins and their various mode of action via common signaling portals to cause toxicity at cellular and organ levels. The large number of known toxins prompted us to select a number of representative species only, which have in common a high specificity towards the nervous system, and share certain discrete signal transduction cascades. Specific examples of these toxins are described in detail including their structural identification, toxic doses, molecular mechanism of action and their potential in translational medicine.

## 2. Toxins and Their Target of Action

### 2.1. Toxins Acting via Plasma Membrane Lipid Domains

#### 2.1.1. Toxins Acting via Pores Formation

A large number of toxins interact directly with lipid components of the cell membrane as prime target for their action [6,7]. The maintenance of cell membrane integrity is a dynamic process regulated by the living cell in order to provide discrete extracellular and intracellular compartments and within the cell, separation into subcellular organelles. A major component of this integrity is the preservation of the ionic gradients necessary for cell function. When a toxin molecule has the potential to perturb the ionic equilibrium, by its insertion into the lipid bilayer and generation of pores, the fine balance of the ion gradients maintenance is perturbed. As a result, changes in the intracellular water content may ensue and cell death may occur. Pore-forming toxins are typically amphipathic polypeptides containing both hydrophilic/polar domain (s) and hydrophobic/non polar domain(s) structures that can vary in size from small peptides to oligomers and up to large macromolecules. Independent of their biological origin, structure or size, the pore forming toxins perform an identical task of permeabilization and/or disruption of the cell plasma membrane (Figure 1, *portal* 1). In the following we will discuss two typical pore-forming toxins and their mode of action, the first is Pardaxin a peptide secreted by *Pardachirus marmoratus* fish (Figure 1 *portal* 1.1) [8]. Pardaxin is a small pore forming peptide toxin, which targets the plasma membrane via hydrophobic/lipophilic interactions with plasma membrane phospholipids and penetrates into the cell membrane. The second is the bacterial *Staphylococcus* α *aureus* toxin which, unlike Pardaxin, forms large size pores in the bilayer [9] (Figure 1, *portals* 1.1 and 7). Pore formation by both toxins lead to a colloid-osmotic process of the cell followed by swelling. While that is a primary event in toxin-cell plasma membrane interaction, pore formation also may trigger secondary events such as Ca^2+^ overload, PLA_2_ activation, eicosanoid production, secretion, endonuclease activation, cytokine release and protein phosphorylation. These all constitute secondary cascades of toxin action which eventually lead to cell death [10]. 

##### 2.1.1.1. Pardaxin

Pardaxin (Figure 1 *portal* 1.1) is an acidic, amphipathic and hydrophobic polypeptide composed of 33 amino acids and a molecular weight of 3.5 kD [11]. It is secreted from the fish glandular epithelial cells together with aminoglycosteroids into the sea water causing toxicity and/or repellency of marine organisms [11]. Pardaxin has been shown to cause excitatory action on neurons through a massive exocytosis of a variety of neurotransmitters [12] including acetylcholine (Ach) at the neuromuscular junction [13], serotonin (5-HT), norepinephrine in cerebral cortex preparations [14] and dopamine in chromaffin and PC12 cells [15,16]. This massive exocytosis may in part account for Pardaxin toxicity. Pardaxin has been sequenced, synthesized and shown to be biologically active, similar to its native counterpart [17,18]. In synthetic liposomes and planar lipid bilayers, Pardaxin can form voltage-dependent pores. These pores behave as hydrated pores for permeant cations and show only modest selectivity for charge [19]. Modelling of Pardaxin pores suggests that they are composed of a cylinder of eight parallel monomers in which right stranded β-barrels are surrounded by eight amphipathic α helices. The shape of the pore is "an hourglass" like structure with a narrow selectivity filter of 4.8 Å diameter. Free charges are absent inside the pore lining, which is consistent with observations that those channels transport both cations and anions [20]. Charge consideration predicts that this cylinder will be inserted into the membrane if the electrical potential on the exoplasmic face of the membrane is positive [19]. Pardaxin is toxic to marine organisms but not toxic (up to 10 mg/kg body weight) to mice or rats upon intravenous (i.v.) injection [21]. Pardaxin is useful for investigating neuronal excitability and neurotransmitter release. It has been used as a voltage-dependent ionophore to affect intracellular ionic composition in studies aimed to clarify the relationship between neuronal depolarization [13] and ion-mediated signal transduction pathways [15]. Since Pardaxin-induced exocytosis of neurotransmitters involves both Ca^2+^-dependent and Ca^2+^-independent mechanisms [13], it has been used in neurochemical studies to investigate specific enzymes associated with the cellular exocytosis [13,22]. Pardaxin activates the arachidonic acid (AA), (C 20:4 n-6), cascade of the cells [22], providing a pharmacological tool to search and develop specific inhibitors of PLA_2_, cyclooxygenase and lipoxygenase enzymes. The ionophore properties of Pardaxin may be exploited for transient permeabilization of cancer cells in order to develop cytotoxic drugs for therapeutic use. The pore forming properties of Pardaxin have been modelled in designing a variety of peptides for different clinical application [23]. Moreover, Pardaxin pores characterization may provide biophysical clues toward a better understanding of the structure and function of eukaryotic ionic channels [24].

##### 2.1.1.2. *Staphylococcus aureus* α-Toxin

*S. aureus* α-toxin (Figure 1 *portal* 1.1) is a 33 kD protein secreted by the most pathogenic strain of *Staphylococcus aureus* bacteria. It binds with both high and low affinity to the membrane of a target cell where it assembles into a relatively large oligomeric pore. *S. aureus* α-toxin was the first pore-forming bacterial toxin to be described, and has since served as an archetype for studies in the field [25,26,27]. Molecular modelling of pore formation including the three-dimensional structure have been elucidated [28]. A mushroom-shaped heptameric α-toxin complex structure, composed from a cap and a stem domain, accompanied by seven rim domains was found. It is approximately 100 Å tall and up to 100 Å in diameter, and the stem domain measures about 52 Å in height and 26 Å in diameter. Contained within the mushroom-shaped homo-oligomeric heptamer is a solvent-filled channel, 100 Å in length, which runs along the sevenfold axis and ranges from 14 Å to 46 Å in diameter. The lytic, transmembrane domain comprises the lower half of a 14-strand antiparallel β barrel, to which each protomer contributes two β strands, each 65 Å long. The interior of the β barrel is primarily hydrophilic, while the exterior has a 28 Å wide hydrophobic belt. The structure proves the heptameric subunit stoichiometry of the α-toxin oligomer and shows that a glycine-rich and solvent-exposed region, within a water-soluble protein, can self-assemble to form a transmembrane pore of defined structure. This unique structure provides an important insight into the principles of membrane interaction and transport activity of β barrel pore-forming toxins [28]. Formation of water-filled non-selective channels permits free passage of ions and low molecular weight molecules (1–4 kDa) such as nucleotides, ATP and mono- and divalent ions such as Ca^2+^. As a result of ATP leakage and loss of the characteristic “milieu interieur” a disruption of essential metabolic processes occurs. Irreversible osmotic swelling and hemolysis of human erythrocytes occur at 1 μM while human platelets swell at 1 nM [26,28]. The LD_50_ of the α-toxin in mice upon i.v. injection is 50 ng/kg [29]. These properties render it as an excellent sensitive tool for controlled permeabilization of the cell plasma membrane. No ionic requirements have been noted for membrane binding or pore formation, and permeabilization can thus be performed in the presence of chelating agents and is most efficient at room temperature. The toxin attaches exclusively to the plasma membrane and does not reach intracellular organelles or cytoplasmic components. Pores created by the *S. aureus* α-toxin do not permit egress of macromolecules from the cytoplasm; hence, enzyme cascades and cellular machineries (e.g., for secretion) remain intact over extended periods. The pores are voltage insensitive, however can be closed in reversible manner in presence of high, non-physiological Ca^2+^ concentrations [26]. Because of controlled manipulation of the intracellular ionic composition, as well as the passage of nucleotides, the α-toxin has been increasingly used as a tool to study exocytosis [26]. Relevant for these applications is the possibility to fine tune the size of the pores and switch them on/off via different stimuli after direct mutagenesis and modifications of the molecule, like opening by a flash of light or by limited proteolysis and closing by Zn^2+^ [27]. *S. aureus* α-toxin is also used as a tool of incorporation of water-soluble sugar trehalose into mammalian calls to protect them from injury [30].

#### 2.1.2. Toxins Mimicking/or Activating Housekeeping Phospholipase A_2_ (PLA_2_)

A large number of organisms including insects, reptiles and mammals, secrete phospholipase A_2_, specifically the soluble form (sPLA_2_), which under certain circumstances, may cause severe damage to the target. PLA_2_ is composed of a large family of housekeeping enzymes, which cleave the sn-2 fatty acid of phospholipids attached to the glycerol backbone. The enzyme is a key player in phospholipid cellular catabolism, primarily in hydrolysis of phosphatidylcholine, to generate lyso-phosphatidylcholine and AA [31]. The family includes the secretory PLA_2_ (sPLA_2_) [32], the cytosolic Ca^2+^ (cPLA_2_)-activated enzyme [33] and several Ca^2+^-independent (iPLA_2_) isoforms [34]. The latter, also called patatin-like phospholipases (PNPLAs), are intracellular enzymes which contain both lipase (GXSXG) and nucleotide-binding (GXGXXG) consensus sequences. Although nine PNPLAs isoforms have been recognized to date, the PNPLA8 (membrane-associated iPLA_2_γ) and PNPLA9 (cytosol-associated iPLA_2_β) are the most widely studied isoforms [33,34]. The iPLA_2_ manifest a variety of activities, are ubiquitously expressed and participate in a multitude of biological processes including lipid metabolism and membrane remodeling, maintenance of mitochondrial integrity, signal transduction, cell differentiation, proliferation and viability [34]. Some of the toxic effects of PLA_2_ have been attributed to the existence of specific receptors for both the venom and the mammalian sPLA_2_ [35]. These sPLA_2_ enzymes are Ca^2+^-dependent and contain a His-Asp motif in the catalytic domain. Individual sPLA_2_ exhibit unique tissue and cellular distribution and provide precursors for pro and anti-inflammatory lipid mediators synthesis and regulate membrane lipid composition. PLA_2_R1, also known as Clec13c belongs to the C-type lectin family and acts as a receptor binding several sPLA_2_ with distinct affinities. PLA_2_R1 exists either as an integral membrane protein with a very large extracellular region comprising of 10 distinct domains and only a short cytoplasmic domain, or as a soluble protein produced by alternative splicing or released from the membrane-bound receptor. PLA_2_R1 may fulfill different roles such as (i) mobilization of sPLA_2_; (ii) transducers of sPLA_2_-dependent signals and (iii) pleiotropic receptors which bind to non sPLA_2_ ligands [32]. Under normal conditions PLA_2_ action on membrane phosphatidylcholine gives rise mainly to AA. The latter is a major precursor for either recycling into phospholipids or for oxidation via alternative pathways catalyzed by cyclooxygenases, lipoxygenases and cytochrome P450-like epoxygenases, generating the corresponding prostaglandins, leukotrienes and thromboxanes, respectively. These compounds also known as eicosanoids, are essential local mediators regulating a large variety of physiological and pathological processes [36] (Figure 1 *portal* 1.2). Snake and bee venoms contain different forms of sPLA_2_ toxins. In the following, we shall discuss three representative members of the toxins abundant in bees and rattle snake venoms. 

##### 2.1.2.1. Bee PLA_2_ Toxin

The bee venom PLA_2_ (Figure 1 *portal* 1.2) belongs to the group III sPLA_2_ and represents up 10%–12% of the dry bee venom content [4]. The enzyme consists of a single polypeptide chain of 130 amino acid residues folded by four disulfide bridges and a glycosyl residue at Asp-13 and has a molecular weight of 15.8 kDa. One of the helices of group III sPLA_2_ enzymes is hydrophobic. It enables binding to the lipid bilayer membrane, while the other helix containing the catalytic domain is non hydrophobic [37]. The bee venom PLA_2_ is a ligand binding and stimulating PLA_2_R1 receptors which mediated the neurotoxic effect. Specific residues in the “interfacial recognition surface” (IRS, which refers to the Ca^2+^ binding loop and 76–91 helix) of PLA_2_ show a high affinity to the PLA_2_R1 receptor, rather than to the plasma membrane lipid bilayer. Studies have shown that PLA_2_ is Ca^2+^-dependent and that the His-34 residue in the catalytic domain functions as a Bronsted base to deprotonate the attacking water molecule, while Asp-64 and Tyr-87 play both a strong supporting role by forming a hydrogen-bonding network with His-34. The latter residues are not critical for catalysis [38]. Studies have demonstrated that mutations that reduce the affinity to PLA_2_R1 receptor also reduce PLA_2_ neurotoxic effects [37] although the enzymatic PLA_2_ activity is retained, supporting the hypothesis that PLA_2_ neurotoxicity its more closely related to its binding affinity for the PLA_2_R1 receptor, rather than to its enzymatic activity [37]. The PLA_2_ from bee venom causes a variety of inflammatory-associated pathologies, including rheumatoid arthritis, septic shock, psoriasis, and asthma [39]. The LD_50_ of bee venom PLA_2_ in mice upon i.v. injection is 0.75 mg/kg [40] and 6.2 mg/kg upon subcutaneous (s.c.) injection [41], causing edema, myonecrosis, prolonged the partial thromboplastin time and reducing the plasma fibrinogen concentration [41]

##### 2.1.2.2. Crotoxin

The venom of the Brazilian rattlesnake *Crotalus durissus terrificus* contains four biologically active proteins. Two of these proteins, Crotoxin (Figure 1 *portal* 1.2), a PLA_2_ enzyme [42] and Crotamine, a cell penetrating peptide [43], act as neurotoxins particularly targeting presynaptic terminals. Crotoxin, a typical snake venom β-neurotoxin, consists of an acidic (three covalently linked polypeptide chains α, 39 β, 35 and γ, 14 residues) and a non-covalently bound basic (122 residues) sub-unit. The overall structure of Crotoxin reveals a unique scaffold for the association of the subunits, with the acidic three-chain subunit rotated by 180° with respect to the main axis and canonical front face orientation of the basic subunit. The individual subunits have little or no toxic effects unless combined [44]. Sequence comparison data suggest that the acidic subunit has been derived from a nontoxic, PLA_2_. When compared with sequences of PLA_2_, the acidic subunit lacks a 22-residue amino-terminal segment and two additional segments that are implicated in phospholipid substrate binding. However, it apparently retains an intact active site, the Ca^2+^-binding loop, and segments involved in subunit binding in homodimeric PLA_2_. The carboxy terminal of the acidic subunit shows strong homology with mammalian neurophysins, lending support to the hypothesis that the acidic subunit functions as a chaperone to prevent nonspecific binding of the toxic basic subunit [44]. Crotoxin inhibits the Na^+^-dependent uptake of norepinephrine, dopamine and serotonin and blocks Ach release from the presynaptic nerve terminal [45]. It alters the anatomy of the nerve cell such as formation of “U” shaped indentations in the axolemma and degeneration of small axons. Furthermore, there is a reduction of the number of synaptic vesicles at the neuromuscular junction. Postsynaptic effects of Crotoxin are due to its binding to the nicotinic cholinergic receptor and prevention of its conformation change, preserving the receptor, like local anesthetic, in a desensitized state [46]. The LD_50_ of Crotoxin is 0.09 mg/kg in mice upon intraperitoneal injection (i.p.) injection [47]. Renal failure is a major cause of death from *Crotalus* venom in humans, whereas in rodents it causes convulsions, massive blood clotting, myonecrosis and cellular respiration failure [47]. Crotoxin-induced cytotoxic effects appear to be highly selective toward cell lines expressing a high density of epidermal growth factor receptors [48] and as a chemotherapeutic agent to achieve refractory solid tumor regression as indicated in phase I clinical trials [49]. Crotoxin is widely used, preferable by encapsulation into liposomes to reduce toxicity, for antivenom production [50]. Crotoxin displays cytotoxic activity against a variety of murine and human tumor cell lines in vitro, which require both the PLA_2_ activity and the ability of the complex to dissociate into their subunits [49]. These finding suggest that Crotoxin has multiple mechanism of actions related to PLA_2_ activity (Figure 1 *portal* 1.2) as well as to the inhibition of ligand gated ionotropic receptors (Figure 1 *portal* 4).

##### 2.1.2.3. Melittin

The principal toxic component in the venom of the honeybee *Apis mellifera*, is Melittin (Figure 1 *portal* 1.2) which comprises about 50% of the dry weight of bee venom. Melittin is a small linear peptide composed of 26 amino acid residues in which the amino-terminal region is predominantly hydrophobic whereas the carboxy-terminal region is hydrophilic due to the presence of a stretch of positively charged amino acids. This amphiphilic property is responsible for its cationic surfactant properties and cytolytic activity [51]. The monomeric Melittin does not contain sPLA_2_ activity by itself. Actually, it facilitates the activation of the endogenous cell housekeeping PLA_2_ activity of the target via rapid binding to the membrane by hydrophobic interaction with zwitterionic phospholipids. This causes disruption of the phosphate group orientation and changes in physical and steric properties of the phospholipid, which eventually weakens the membrane barrier function [51]. Melittin facilitation of PLA_2_ activity stems from its ability to dissolve into the hydrocarbon region of the bilayer, the subsequent formation of melittin-phospholipid domains and consequent alteration of the membrane surface resulting in antibacterial and hemolytic activity. The highly charged C region and the amino phosphate dipole has a strong electrostatic interaction that results in membrane perturbation, rendering the lipid target susceptible to PLA_2_ cleavage. Upon i.v. or s.c. injection in mice, the LD_50_ of Melittin is 2.8 mg/kg [40] and 20.8 mg/kg [41], respectively. Bee venom PLA_2_, but not Melittin, has been shown to be a strong inducer of IgE antibodies production and is the major anaphylactic agent in the venom. While Melittin and PLA_2_ exhibit distinct toxic effects on biological systems, there is also evidence of a synergistic action [52]. Melittin has no effect on cPLA_2_ [53,54]. Melittin has been an important biochemical tool to stimulate PLA_2_ in various cells and tissues, such as rat anterior pituitary cell cultures, human erythrocytes, anterior pituitary tissue, pancreas, rat adenohypophysis, vascular endothelial cells, human leucocytes, platelets [55] and neurons [54]. Melittin has been used as a neurochemical tool to investigate the role of sPLA_2_ in modulating synaptic transmission [54]. It has also been widely used as a component of anti-venom and for desensitization therapy against bee stings [56]. Cytotoxic and membrane penetrating properties of Melittin have rendered it useful in cancer therapy, either as an active ingredient or as an absorption enhancer [57]. Melittin has proven itself as a potent antimicrobial agent that may be used for the treatment of eye infections [58]. Recently some evidence was provided that Melittin inhibits HIV-1 gene expression at the transcriptional level and therefore may be used for anti-AIDS therapy [59].

### 2.2. Toxins Acting via Ion Channels

Ion channels are important portals for a variety of toxins interacting with the plasma membrane [60]. Under normal conditions, the physiological role of these channels is to regulate with high selectivity, the transfer of cations or anions in order to maintain a resting membrane potential and to control action potentials in excitable tissues. There are many different types of ion channels, classified by their gating characteristics and by the type of ion passing through. These channels very effectively transport ions in a time frame period of milliseconds, often rising to 10^6^ ions per second or greater. In view of the importance of ionic gradients for maintaining neuronal, cardiac, skeletal or smooth muscle tissue functions, numerous toxins, which modulate ion channel conductance and/or kinetics by serving as either channel openers or blockers with different selectivity, have been investigated. In non-excitable tissue, ion channels regulate important biological functions such as epithelial transport of nutrients and ions, T-cell activation in inflammatory processes and release of insulin by pancreatic β-cells. It has been well established that most Na^+^, K^+^, Ca^2+^ and some Cl channels, are voltage-gated [61] but others including certain K^+^ and Cl^−^ channels, transient receptor potential (TRP) channels, ryanodine receptors and IP_3_ receptors, are relatively voltage-insensitive and are gated by second messengers and other intracellular and/or extracellular mediators [62]. Voltage-gated ion channels (Figure 1 *portal* 2) open or close depending on the voltage gradient across the plasma membrane. Ions are conducted through the channels down their electrochemical gradient, which is dependent on ion concentration and plasma membrane potential, without the need of ion pumps (Figure 1 *portal* 3). These ion channels at large are selectively targeted by certain toxins contained in dinoflagellate [63] , plant [64] cone snail [65], scorpion [66] and snake venoms (Figure 1 *portal* 2) [67]. 

#### 2.2.1. Voltage-Gated Potassium Ion Channels (Kv)

The voltage-gated K^+^ channels are transmembrane channels specific for K^+^ ions (Figure 1 *portal* 2) and are sensitive to voltage changes in the membrane potential [68]. These channels are known mainly for their role in repolarizing (voltage-dependent Kv) the cell membrane following action potentials, which regulate cellular processes such as Ca^2+^ signaling, cell volume, secretion, proliferation and migration [69]. Kv channels usually have a homo-tetrameric structure. The transmembrane domain, the α-subunit, consists of six helices (S1–S6) forming two structurally and functionally different parts of the tetrameric channel: a potassium ion-conducting domain (pore domain), helices (S5–S6) located in the channel center and a domain sensible to changes in the membrane potential (voltage-sensing domain, VSD), helices (S1–S4) located on the channel periphery. A large number of polypeptide ligands of Kv channels have been found in the venoms: bees (Apamin and Tertiapin, targeting mostly K_Ca_ and K_ir_, respectively), snakes (Dendrotoxins targeting Kv channels with different selectivity), sea anemones (AeK, BgK, HmK, ShK, AsKC1, APEKTx1, BDS-I, Am II, APETx1 polypeptides targeting Kv 11, Kv 3.4, K_Ca_ channels), cone snails (κ-Conotoxins targeting Kv and K_Ca_ channels with different selectivity), spiders (Hanatoxin (HaTx1) targeting Kv2.1; HpTx1 ,PaTx1 targeting Kv4; ScTx1, HmTx1 targeting Kv2 and Kv4 etc.), and scorpions (Charybdotoxin and Kaliotoxin, targeting K_Ca_, Margatoxin targeting Kv1.3 and Iberiotoxin targeting Kv1.3 and K_Ca_,) etc. In the following paragraphs, several dendrotoxins with nanomolar affinity to Kv channels, that have become classical tools, are discussed.

##### Dendrotoxin

Dendrotoxins (Figure 1 *portal* 2) constitute a family of toxins that block voltage-dependent K^+^ channels in excitable cells [70]. These toxins were originally isolated from the venom of three species of the African mamba snakes; the eastern green mamba (*Dendroaspis angusticeps*), the western green mamba (*D.viridis*) and the black mamba (*D. polylepis*) [71]. The dendrotoxins are small basic proteins consisting of 57–60 amino acids organized in a single polypeptide chain cross-linked by three disulfide bridges [71]. Six natural variants have been characterized: two isoforms of α-dendrotoxin and δ-dendrotoxin from *Dendroaspis angusticeps*, toxin I and toxin K from *D. polylepis* and Dv-14 from *D. viridis* [72,73]. Two related toxins, β- and γ-dendrotoxin, from *D. angusticeps* have been partially sequenced [74]. Dendrotoxins were discovered due to their ability to facilitate the release of the neurotransmitter Ach at the neuromuscular junction [71,75]. This effect is the result of their ability to block certain voltage-dependent K^+^ channels in nerve presynaptic terminals [74,76,77]. Dendrotoxins block neuronal Kv channels, and often induce “repetitive action potentials”. In studies of cloned K^+^ channels, dendrotoxins preferentially block K_v_1.2 (IC_50_ 0.1–17 nM), K_v_1.1 (IC_50_ 1–20 nM), and K_v_1.6 (IC_50_ 52 nM), with little effect on other K_v_ isoforms [78,79]. Unlike β- and γ-dendrotoxins the α- and δ-isoforms block different K^+^ currents in synaptosomes [74] giving rise to the possibilities that different subtypes of voltage-dependent K^+^ channels express different sensitivities to individual dendrotoxins. The toxins have been used to isolate K^+^ channels subunits proteins from brain membranes [80,81] and in brain slices to quantify the distribution of K^+^ channels in different regions [82,83]. Dendrotoxins are useful pharmacological tools because of their high potency and selectivity for different Kv [70]. The lethal dose 50% (LD_50_) is about 20–25 μg/g upon i.v. injection in mice [72]. The α-dendrotoxin lethal potency is much higher upon direct injection into the rat brain with median lethal dose (MLD) in the range of 2.5 ng/g.

#### 2.2.2. Voltage-Gated Sodium Channels (Na_v_)

The voltage-gated Na^+^ channels Na_v_ (Figure 1 *portal* 2) are a family of at least 9 members which are largely responsible for action potential generation and axonal conduction. The pore-forming α subunit(s) of the Na_v_ are very large and reach up to 4000 amino acids. They consist of four homologous repeating domains (I–IV) each comprising six transmembrane segments (S1–S6) and a total of 24 transmembrane segments. The members of this family also interact with auxiliary β subunits [84]. Na_v_ are targeted by a large variety of chemically distinct toxins, produced by animals and plants. At list six distinct class of Na_v_ neurotoxins have been categorized on the basis of the physiological activity and binding site. The first group consisting of Tetrodotoxin, Saxitoxin and μ-Conotoxin bind and block the pore of the channel and identify Na_v_ site I. The second group consisting of Batrachotoxin, Veratridine, Aconitine and Grayanotoxin cause persistent-enhanced activation, and block of activation and identify Na_v_ site II. The third group consisting of α-scorpion toxins and sea anemone toxins II and Atrachotoxins caused slowed inactivation and identify Na_v_ site III. The fourth group consists of β-scorpion toxins which shift voltage dependence of activation and identify Na_v_ site IV. The fifth group consists of Brevetoxins and Ciguatoxins which shift voltage dependence of activation and block inactivation and identify Na_v_ site V. The sixth group represented by δ-Conotoxin causes slowed inactivation and identify Na_v_ site VI [85]. Na_v_ site III targeted by *α* scorpion toxin [66] detailed below, exemplify the mode of slow inactivation attack of Na_v_ to suppress neuronal excitability. 

##### α-Scorpion Toxin

The α-scorpion (Figure 1 *portal* 2) toxins are a potent group of Na_v_ channels blockers composed of 60–76 amino acid residues, arranged in a common three-dimensional structure formed by an α-helix and three strands of β-sheet structure motifs, cross-linked by four disulfide bridges [86]. The α-scorpion toxin binds to an extracellular site of the sodium channel α -subunit, receptor site 3, and induces prolonged action potentials in excitable tissue by inhibiting transitions of the channel from the open to the closed states [87,88]. Scorpion α-toxins are classified as neurotoxins with high selectivity to mammals and/or other species [89]. The scorpion α-toxin binding to a sodium channel is voltage-dependent [85] and associated with the voltage-dependent gating transition process [90]. A proposed molecular model for its mechanism of action suggests binding to Na_v_ in the resting state and inhibition of fast inactivation by interaction with a receptor site formed by domains I and IV [90]. Thus, toxin binding prevents the conformational change required for fast inactivation, slows down or prevents channel inactivation, thereby enhancing excitation. Therefore, α-scorpion toxins are defined in molecular terms as “gating modifier” toxins [91]. The LD_50_ of α-scorpion toxin is in the range of 0.05 mg/kg upon subcutaneous injection (s.c.) [92] to 0.09 mg/kg upon intracerebroventricular injection (i.c.v.) in the mouse [93]. Due to their high affinity, specificity and different molecular mechanisms by which various toxins modulate sodium channel subtypes action, they have been useful tools to investigate the sodium channels structure and their electrophysiological properties, as well as in drug discovery for novel antiarrhythmic therapeutic molecules. As sodium channel modulators, they also exerted a therapeutic potential in treating diseases such as epilepsy, neuropathic pain, congestive heart failure and arrhythmia [87].

#### 2.2.3. Voltage-Gated Calcium Channels (VGCC)

Voltage-gated Ca^2+^ channels are a family of 10 members, (Figure 1 *portal* 2) which mediate Ca^2+^ entry into the cells in response to membrane depolarization [94]. They are divided into low threshold voltage (T- type) and high threshold voltage (L, N, Q/P, R type) classes. The high-voltage Ca^2+^ channels consist of a pore-forming α1 subunit, transmembrane, disulfide-linked complex of a α2 and δ subunit, an intracellular β subunit and in some case a transmembrane γ subunit. These voltage-gated Ca^2+^ channels play an important role in both linking muscle-electrical excitation-coupling with the mechanical contraction, as well as neuronal-excitation-coupling with neurotransmitter release. The α subunits have an overall structural resemblance to those of the sodium channels and are equally large. Venoms from fish hunting mollusks, scorpions, snakes and spiders contain a large spectrum of toxins that include blockers of VGCC. These toxins act by two principal mechanisms: physical occlusion of the pore and prevention of activation gating. Many of the VGCC toxins have evolved to occupy with high affinity their binding pocket on the VGCC pore rendering VGCC block poorly reversible. Moreover, several of the best-characterized VGCC’s blocking toxins have developed a high degree of channel subtype selectivity. These toxins include for example the snake venom’s calciseptine, calcicludine, taicatoxin as well as the cone snail Ω-conotoxin [95] and funnel-web spider venom Ω-agatoxin [96] the properties of which are discussed below. 

##### 2.2.3.1. Ω -Conotoxin

The Ω-conotoxins (Figure 1 *portal* 2) are small peptides (24–27 amino acids) isolated from the marine cone snail venom. The peptides retain a highly conserved six cysteine framework pattern which forms three disulfide bonds (between Cys 1–4, Cys 2–5 and Cys 3–6) forming a 4-loop structure. Loops 2 and 4 are instrumental in directing the Ω-conotoxin selectivity, while loops 1 and 3 have little effect [94,97]. A C-terminal amide, may limit potential degradative carboxylase activity, which would otherwise render the peptide biologically inactive [98]. The identical disulfide arrangement and a conserved Gly in loop 1 are insufficient to define VGCC selectivity, but instead provide a structural platform that allows the four hypervariable loops to display the functional groups that are necessary for interaction with VGCC [94]. The family contains different neurotoxic peptides derived from *Conus geographus* (GVIA), *Conus magnus* (MVIIA, MVIIC, and MVIID) and *Conus catus* (CVID) which inhibit voltage-gated Ca^2+^ channels (VGCCs). GVIA and MVIIA are highly specific for N-type Ca^2+^ channel, while MVIIC, preferentially targets P/Q channels [26]. Conotoxins are known as ‘shaker peptides’ as they induce persistent tremors in mice upon intracerebral injection [99]. MVIIA and GVIA cause tremors at low doses, but not death at higher doses, upon intracranial injection into mice. These findings reflect the differential distributions of VGCC in the central as opposed to the peripheral nervous system. Upon intracranial injection, a progressive respiratory distress is observed in mammals prior to death, while intrathecal administration of the conotoxins CVID, GVIA, MVIIA (LD_50_ = 3.5, 0.6, and 0.68 µg/kg, respectively) cause moderate to severe toxicity [100]. Administration of these peptides in lower vertebrates cause prompt death [101]. The N-type calcium channel is coupled by a GPCR to the opiate receptor, which generates pain by increasing intracellular Ca^2+^ in sensory neurons. Therefore, blocking N-type Ca^2+^ channels by MVIIA conotoxin in sensory pain neuronal pathways, has been used successfully to block pain [102]. A synthetic form (Ziconotide, Prialt) of MVIIA has been introduced into the clinic for pain therapy [103]. The latter is a new, non-opioid, non-addictive treatment for chronic pain and is >10 fold more potent than intrathecal administered morphine [104,105].

##### 2.2.3.2. Ω-Agatoxin

Several toxins isolated from the *Agelenopsis aperta* funnel-web spider venom, represent a diverse group of polypeptide Ca^2+^ channel blockers, which interfere with neurotransmitter release and synaptic transmission [106]. There are four types of Ω-agatoxins (Figure 1 *portal* 2): (i) Aga-IA, is a heterodimeric peptide which blocks presynaptic Ca^2+^ channels at the neuromuscular junction and Ca^2+^ action potentials in neurosecretory neurons of insects [106,107]; (ii) Aga-IIA is a large monomeric polypeptide which blocks presynaptic Ca^2+^ channels and mammalian cardiac L-type Ca^2+^ channel currents without affecting gate currents in diverse species such as insects [106], birds [108] and mammals [109,110]; and (iii) Aga-IIIA, a monomeric peptide which blocks presynaptic Ca^2+^ channels in birds [108] and N-type, L-type, and P-type channels in mammals with equal potency [111,112]. Intracerebroventricular (i.c.v) injection of Aga-IIIA into young mice causes convulsions and death. Aga-IIIA has been used to characterize low-threshold T-type currents in atrial myocytes as it selectively blocks high threshold L-type currents in these cells [113]. This toxin has advantages over dihydropyridines, the common drugs used to block L-type calcium channels, in that the blockade occurs over a wide spectrum of voltage. T-type currents also are susceptible to dihydropyridines under certain conditions [114], but are completely resistant to inhibition by Aga-IIIA. Another useful and characteristic property of Aga-IIIA is the complete inhibition of L-type currents in atrial myocytes without affecting gating currents [110]. Application of the toxin has revealed that significant portions of ionic currents under physiological calcium conditions are masked by gating current. Similarly, gating currents also mask the kinetics of L-type channel activation and deactivation. The toxin therefore, provides a valuable tool to characterize the kinetics and relative contributions of ionic and gating currents to total currents recorded in myocytes and neurons. The type IV group agatoxins, Aga-IVA and Aga-IVB, are monomeric peptides containing four internal disulfide bonds [109,115]. Both are highly specific antagonists of the P-type calcium channel at low concentrations (Kd = 3 nM) in the mammalian brain [109]. At higher concentrations (>100 nM), Aga-IVA blocks Q-type calcium currents in culture of rat cerebellar granule neurons, resulting in suppression of synaptic transmission [116]. By blocking P-type calcium channels through modification of voltage-dependent gating [115,117], the toxin shifts the activation of the channel to positive potentials that are normally not reached during physiological activity of the neuron. The toxin therefore, raises the energy barrier for voltage-dependent gating to non- physiological levels. Aga-IVA provides a pharmacological probe that, in combination with other subtype-specific toxins, has been useful in discriminating the roles of calcium channels in neurotransmitter release in the CNS. Soon after its discovery, it was used to demonstrate that P/Q-type channels are coupled to glutamate release from cortical synaptosomes, and that both N- and P-type channels are jointly involved in glutamate and dopamine release from striatal synaptosomes [118,119]. Similar interpretations were made from electrophysiological experiments [111,120,121,122]. Overall, these studies illustrate the utility of subtype-specific omega toxins probes in defining the types of receptors and ion channels that are involved in neuronal function.

#### 2.2.4. Chloride Channels (CLCs)

Chloride channels (Figure 1 *portal* 2) represent a superfamily of channels consisting of a dozen of members involved in many cellular functions including cell volume regulation, muscle contraction, secretion, and modulation of neuronal signal transduction. Under physiological conditions the passive flux of Cl^−^ anions across biological membranes is regulated by events such as ligand binding, changes in intracellular Ca^2+^ and membrane potential [123]. These channels are non-selective for small anions; however, they conduct mainly Cl^−^ ions. The channels can be classified, due to their structural characterization, into four groups: (i) ligand-gated receptor Cl^−^ channels, (ii) cAMP-regulated channel cystic fibrosis transmembrane conductance regulator (CFTRs), (iii) voltage gated Cl^−^ channels (CLCs) and (iv) Ca^2+^-activated Cl^−^ channels. 

##### Chlorotoxin (CTX)

Chlorotoxin (Figure 1 *portal* 2) is a 36 amino acid long neurotoxic peptide purified from the *Leiurus quinquestriatus* scorpion venom. Its molecular weight is 3.9 kDa and contains 8 cysteines. Chlorotoxin has been the first high affinity ligand of the CLC reported [124,125,126]. CTX belongs to the short-chain toxin family of proteins, specifically blocking the small conductance epithelial chloride channels. Structural analysis of CTX in solution, revealed a small three-stranded antiparallel β-sheet packed against an α-helix, thereby adopting the same configuration as Charybdotoxin and other members of the short α-scorpion toxins family. There are four disulfide bonds in CTX conferring a high degree of structural stability [125]. Each CLC can be blocked effectively by 0.2 mg/mL CTX indicating that only one ligand is bound per one chloride channel [126]. An LD_50_ value of 4.3 mg/kg was determined in mice upon i.v. injection of CTX analog [127]. Voltage-dependent CLC which are up regulated in glioblastoma tumors, are blocked by CTX [128]. BmKCT, a CTX analog isolated from the scorpion *Buthus martensii karsch* venom, reduces the chloride currents by 75% [129]. CTX is non-toxic to mammals, however in crayfish, CTX causes a loss of motor control promptly after injection of 1.23–2.23 μg/g which progresses to a rigid paralysis [126]. Because of its specificity and selectivity for glioma cells, CLC, the synthetic version (TM-601) of CTX, has been engineered in *E.coli* and by chemical synthesis [130]. The peptide has been radiolabeled with radioactive iodine (^131^I) to obtain ^131^I-TM601 and has been used for imaging and radiotherapy of malignant glioma [131,132]. The iodine-131-TM-601 peptide was evaluated in phase I clinical trials for the treatment of solid tumors or recurrent high-grade gliomas in humans [133]. ^131^I-labeled multifunctional preparations of CTX and analogs may offer in the clinic important diagnostic tools for imaging and radiotherapy of human glioma tumors enriched in CLCs [134].

### 2.3. Toxins Acting via Ion Pumps

The term ion pump encompasses all the transporters, which are capable of performing active transport. Ion pumps that use ATP and contain an ATPase which hydrolyzes it, are called ATP-driven primary pumps [135]. They can be divided into three major subgroups: P-type ATPases; F-type or V-type ATPases and ATP-binding cassette transporters [136]. The P-type ATPases are multimeric proteins, which transport (primarily) inorganic cations. The F-type or V-type ATPases, are proton-coupled motors, which can function either as transporters or as motors. Finally, the ATP-binding cassette represents a group of transporters dedicated mainly to drug disposition as well as clearance of endogenous hydrophobic solutes [136,137]. Secondary pumps exploit the energy stored in ion (often Na^+^) electrochemical gradients to drive the transport of another substrate against its concentration gradients. Therefore, these pumps perform as co-transporters or counter- transporters or exchangers, depending on the relative direction of the ion flow [135]. 

#### 2.3.1. Na^+^/K^+^ ATPase

The most widely studied ion pumps are: (i) the Na^+^/K^+^ ATPase which is a house-keeping enzyme ubiquitous in all animal cells which maintains the electrochemical potential gradients, of Na^+^ and K^+^ ions across the cytoplasmic membrane; (ii) the Ca^2+^-ATPase of the sarcoplasmic reticulum (SR Ca-ATPase) which pumps Ca^2+^ ions back into the lumen of the sarcoplasmic reticulum (SERCA) causing muscle relaxation by reducing the cytoplasmic Ca^2+^ to concentrations less than 0.1 μM; (iii) and the gastric H^+^, K^+^-ATPase which is expressed in parietal cells of the gastric mucosa where it drives the hydrochloric-acid secretion into the gut lumen by active transport of H^+^ ions, followed by passive transport of Cl^−^ anions. The Na^+^/K^+^ ATPase, is an energy-transducing ion pump consisting of two types of subunits, designated α and β, in addition to a single-transmembrane -spanning protein, FXYD named for the conserved amino acids in its signature motif: (Phe-Xxx-Tyr-Asp). The α subunit, responsible for binding Mg^+2^, ATP, Na^+^, K^+^ and the cardiac glycosides toxins, is considered the catalytic subunit of the enzyme. The β subunit is a glycoprotein that seems to act as an adhesion molecule regulating gap junction proteins. It is involved in structural and functional maturation of the holoenzyme and it facilitates transport and maintenance of the α subunit in the plasma membrane [138,139,140]. The FXYD protein regulates enzyme function, thus adapting the kinetic properties of Na^+^ and K^+^ transport to the physiological needs of the cell [141,142]. Four α subunit variants, as well as three β, and seven FXYD subunit variants have been identified [138]. The well-established function of Na^+^/K^+^ ATPase is to use ATP as the prime energy source to drive excess Na^+^ out of cells in exchange for K^+^, thereby maintaining an essential ionic and osmotic balance. The Na^+^/K^+^ ATPase is tagged as a “signalosome” complex of multifold cellular tasks [138,143] therefore toxins targeting this signal *portal* significantly impair ion homeostasis as well as other basic functions of the cell. Below an account of the plant-derived cardiac glycosides which target the Na^+^/K^+^ ATPase is presented. Several toxins such as Thapsigargin [144] blocking SERCA and the *Helicobacter pylori* bacterial toxin [145] blocking gastric H^+^, K^+^-ATPase, are beyond the scope of this review.

##### Cardiac Glycoside Toxins

Cardiac glycosides (Figure 1 *portal* 3) are a group of steroid-like compounds that inhibit ATP binding and disrupt the ability of the Na^+^/K^+^ ATPase to perform Na^+^/K^+^ exchange in an efficient manner. This, in turn, results in an enhanced entry of Ca^2+^ ions into cells, leading under toxic conditions to calcium overload followed by cell death. Cardiac glycosides originate from different plants and are widely used in therapy of congestive heart failure and arrhythmia. Typical compounds extracted from *Foxglove* and *Oleander* plants include cardenolides, such as digitalis, digoxin, and oleandrin [146,147]. The mechanism of their action for the treatment of congestive heart failure arises from the selective binding and inhibition of Na^+^/K^+^ ATPase, with a resulting increase in intracellular myocardium Ca^2+^ concentration required for heart mechanical activity. Cardiac glycosides being toxins however, have a narrow therapeutic index. 

Ouabain, the most common cardiac glycosides, binds to the Na^+^/K^+^ ATPase and triggers several intracellular signaling cascades involving many intracellular components [143,148,149]. These signaling pathways are affecting cellular physiology through the activation of protein kinase Src, transactivation of the epidermal growth factor receptor (EGFR), activation of Ras and mitogen-activated protein kinases (MAPKs) and increased generation of reactive oxygen species (ROS) in the mitochondria. Activation of these cellular pathways is also linked with translocation of Na^+^/K^+^ ATPase, through endocytosis, to the nucleus [138,150]. The toxicity (LD_50_) of cardiac glycoside is about 10 mg /kg upon i.p. injection in mice, [151]. Development of clinically relevant cardiac glycosides could be helped by systematic evaluation of the large family of cardiac glycosides and synthetic analogs available to date. Further development of synthetic, semi-synthetic, or naturally occurring cardiac glycosides, with assessment of their toxicity and structure-activity relationships, might expand the possibilities of developing a cardiac glycoside with a wider therapeutic index [150]. 

### 2.4. Toxins Acting via Ligand-Gated Ionotropic Channel Receptors

Ligand-gated ionotropic channel (LGIC) receptors are part of a discrete group of ion channels (Figure 1 *portal* 4) which opens and conduct ions in response to specific ligands [152]. Ion flux is a passive process driven by the electrochemical gradient for the permeant ions. Ligand binding causes a conformational change in the structure of the receptor protein that ultimately leads to the opening of the channel, to enable subsequent ion flux across the plasma membrane and elicit depolarization. Modulation of gating can occur by binding endogenous or exogenous allosteric modulators. In the CNS and at the neuromuscular junction, LGICs mediate fast synaptic transmission, in millisecond intervals. Such transmission involves the release of a neurotransmitter from the pre-synaptic site and the subsequent activation of post-synaptic receptors that mediate a rapid, phasic, electrical signal (excitatory or inhibitory post-synaptic potential). In addition to their traditional role in phasic neurotransmission, some LGICs mediate a tonic form of neuronal control that results from the activation of extra-synaptic receptors. Some LGICs are expressed by non-excitable cells suggestive of additional functions. Examples of such channels include the cation-permeable, nicotinic Ach receptor [152], targeted by many snake toxins such as the three-fingers toxin α-Bungaratoxin [153] and ionotropic glutamate-gated receptors [154], targeted by Domoic acid [155] and Kainic acid [156] (Figure 1 *portal* 4).

#### 2.4.1. α-Bungarotoxin (α-Bgtx) 

α-Bgtx (Figure 1 *portal* 4) is a powerful toxin derived from the venom of the elapid snake Taiwanese banded krait *Bungarus multicinctus* which acts as an antagonist of the ligand-gated ionotropic nicotinic cholinergic receptor [157]. α-Bgtx is a single polypeptide containing 74 amino acids (molecular weight 8 kDa), cross-linked with five disulfide bridges. To date more than 80 α-neurotoxins (or postsynaptic neurotoxins) have been identified and some prepared in a recombinant form [158]. These α-neurotoxins have been classified into two distinct subclasses: the “short” toxins that all contain 4 disulfide bridges and 60–62 amino acids, and the “long” toxins with five disulfide bridges and 66–74 amino acids [159]. The X-ray structure [160] and two-dimensional nuclear magnetic resonance (NMR) characterization, indicate that the toxin has a three-loop structure and apart, from the carboxy-terminal tail, it is a relatively flat molecule with three main extended loops roughly in one plane and covering an area of about 40 X 30 Å with a depth of about 20 Å. A number of residues are important in the binding of α-Bgtx to muscle nicotinic Ach receptors (AChRs), consistent with the hypothesis that multi-point interactions take place between the toxin and the AChRs [161,162]. α-Bgtx binds with high affinity and specificity to the postsynaptic skeletal muscle nicotinic Ach receptors at the α-subunit of the receptor and to some types of neuronal AChRs, preventing their activation by ACh. The binding of the toxin occurs at close proximity to a sulfhydryl group, thus focusing attention to the 4 cysteine residues present in the extracellular portion of the α-subunit. α-Bgtx does not cross the blood-brain barrier [163]. The toxicity (LD_50_) of α-Bgtx from different snake species upon i.v. injection in mice, is in the range of 0.2–1.2 mg/kg [164]. Clinical signs of intoxication include respiratory distress and severe neuromuscular paralysis. Death occurs in a period of 30 min to 4 h after exposure. Due to its high affinity to the different α subunits of nicotinic AChRs (K_d_ 10^−12^–10^−9^ M), α-Bgtx has been used as a ligand to identify, localize, quantify and isolate AChRs present in detergent-solubilized Torpedo electric organs and mammalian muscle and brain tissue. These functionally, purified and reconstituted AChRs made it possible to obtain the partial amino acid sequence of the AChR subunits, which subsequently were used as probes for cloning of the complete cDNA and genomic sequences of the muscle AChRs subunits [162]. It has been demonstrated by cloning and functional expression of the α7, α8 and α9 subunits in oocytes [165,166] that the α subunit of AChRs is the target of α-Bgtx. Reconstituting affinity purified α-Bgtx receptors in artificial lipid bilayers [167], and electrophysiological recordings from neurons in culture [168] also demonstrated the high affinity of the toxin to the AChR. Autoradiography of α-Bgtx is a useful method in characterization of regional distribution of nicotinic receptor sites in cholinergic synapses of the CNS [163]. This toxin can be used towards development of novel antagonists of nicotinic AChRs which may be used in the clinic as drugs to relax the skeletal muscle before surgical procedures [169].

#### 2.4.2. Domoic Acid (DA) Toxin

Domoic acid (Figure 1 *portal* 4) is an excitatory neurotoxin of ligand-gated ionotropic glutamate receptor produced by the red algae *Chondria armata* [170]. Under favorable environmental conditions, algae overgrow, and create a bloom known as “red tide” which is poisonous to humans upon consumption of fish which fed on these algae. The DA has a molecular weight of 311.34 and its structure is 2S-2, 3β, 4β (1Z, 3E, 5R) -2-carboxy-4-(5-carboxy-1-methyl-1,3 -hexadienyl)-3- pyrrolidineacetic acid. It is water-soluble and is a structural analogue of proline. DA is an excitatory amino acid containing the structure of the excitatory neurotransmitter glutamate. Three isomers of DA (D, E, and F) of little biological activity have been described [171]. Being a rigid analog of this neurotransmitter, DA is a potent agonist of kainate subtype glutamate receptors [172]. Its structural rigidity leads to a tight binding to the receptors causing its excitatory effect to be 30 to 100 times more potent than glutamate itself [173,174]. DA activates non-NMDA receptor subtypes in the hippocampus [175] and induces Ca^2+^ overload which is responsible for the high toxicity observed in vitro and in vivo [176]. DA toxicity (LD_50_) to mice upon i.p. injection is 3.6 mg/kg [177]. Administration of DA and its analogues in rodents and monkeys cause limbic seizures, memory deficits and neurodegeneration. In humans, consumption of DA due to contaminated seafood causes gastrointestinal symptoms within 24 h followed by nausea, vomiting, headache, diarrhea, or abdominal cramps. Neurological symptoms reported in humans and animal models include confusion, memory loss, disorientation, seizures and coma [178,179,180].

#### 2.4.3. Kainic Acid (KA) Toxin

Kainic acid (Figure 1 *portal* 4) is a low molecular weight pyrolidine derivative that possesses two carboxylic acid moieties and an isopropanol side chain. It is a well-known toxin that acts as antagonist of the ligand-gated ionotropic glutamate receptor (iGluRs). The molecule, a rigid analog of glutamate, was first isolated from the seaweed *Digenea simplex* and is 30 to 100-fold more potent than glutamate as a neuronal excitant [181,182]. The molecule is a strong excitatory neurotoxin, that following systemic or intracerebral administration generates seizure in rodents. The targets of KA, the iGluRs, are classified into three subtypes according to the compounds that preferentially activate them: AMPA (2-amino-3-hydroxy-5-methylisoxazole propionic acid), NMDA (*N*-methyl-d- aspartate) and KA [62]. Within these subclasses, multiple subunits have been identified. These subunits can form homomeric and heteromeric assemblies of functional iGluRs. Functional kainate receptors can be expressed as homomers of GluK1, GluK2 or GluK3 subunits. GluK1-3 subunits are also capable of assembling into heterotetramers. Two additional kainate receptor subunits, GluK4 and GluK5 form high affinity binding sites for kainate, when individually expressed, but lack functions. However, they can form heteromers when expressed with GluK1-3 subunits. Kainate receptors may also exhibit “metabotropic” functions. As found for AMPA receptors, kainate receptors are modulated by auxiliary subunits. An important functional difference between AMPA and kainate receptors is that the latter require extracellular Na^+^ and Cl^−^ ions for their activation [62]. However, KA also activates the AMPA receptors, which are composed of subunits GluR1-4, even if with lower affinity [183]. The presence of functional receptors selective for KA in hippocampal neurons was demonstrated [184]. KA neurotoxicity is mainly caused by the activation of KA receptors, but also by certain AMPA receptors. Systemic or intracerebral administration of KA in adult rats cause persistent seizures and seizure-mediated brain damage syndrome, involving selective degeneration of neurons, especially in the striatal and hippocampal areas of the brain [182]. CA3 pyramidal neurons are amongst most vulnerable to KA-induced toxicity in the brain [185], and KA injections cause epileptiform seizure in the CA3 region of the hippocampus. These seizures propagate to other limbic structures and are followed by a pattern of cell loss that is similar to that seen in patients suffering from temporal lobe epilepsy [186]. The cellular mechanisms underlying KA toxicity are not fully understood, and yet, a line of evidence relates the KA neurotoxicity to arachidonic acid release (Figure 1 *portal* 1.2). KA-induced neurotoxicity involves stimulation of phospholipase A_2_ (PLA_2_), release of AA, accumulation of lipid peroxides, changes in prostaglandin metabolism, decline in reduced glutathione level and accumulation of 4-hydroxy-nonenal, a potent neurotoxic end-product of lipid peroxide decomposition [187,188]. The channel conductivity of KA and AMPA receptors is mainly for Na^+^, and therefore excessive stimulation of KA receptors may produce membrane depolarization, accompanied by elevation in intracellular Ca^2+^ levels, probably via NMDA receptors. The increase in the latter may stimulate PLA_2_ directly or regulate some other transduction mechanisms leading to the activation of PLA_2_ activity [188]. The KA toxicity (LD_50_) in mice upon i.v. injection is 35 mg /kg [189]. The doses of KA that generate seizures are 30 mg/kg for the adult and 2 mg/kg for the young mouse [190]. KA is a very useful tool for the study of glutamate-driven synapses in general and glutamate receptors in particular. KA-induced epilepsy in mice is used today as a classical model for human temporal lobe epilepsy studies [185].

### 2.5. Toxins Acting via G-Protein Coupled Receptors (GPCRs)

G-protein coupled receptors share a common architecture, each consisting of a single polypeptide with an extracellular N-terminal, an intracellular C-terminal and seven hydrophobic transmembrane helical protein domains (7 TM) linked by three extracellular and three intracellular loops. Nearly 800 GPCR’s have been identified to date in humans [191], of which more than 50% exhibit sensory functions including olfaction (~400), taste (33), light perception (10) and pheromone signaling (5). The remaining 350 GPCR’s account for non-sensory GPCR’s which mediate cellular signaling by ligands that range in size from small lipid molecules to peptide and large proteins. The seven trans-membrane segments of the receptor (7TM) are commonly used interchangeably with “GPCR”, although there are some receptors with seven transmembrane domains that do not signal through GTP binding proteins. Following completion of cloning of many of these receptors a thorough screening of various snake venoms that may target specific GPCR was undertaken [192]. In the course of these studies, several new receptor ligands have been identified, that show subtype selective properties, not generally achieved by chemical design. The mamba snakes for example have been a rich source for GPCR ligands studies. The typical GPCR's are muscarinic cholinergic receptors targeted specifically by different toxins with representative example of the mamba snake *Dendroaspis muscarinic toxins* (Figure 1 *portal* 5) [193], which by inhibiting muscarinic receptors may cause pathological effect in the tissue innervated by the parasympathetic cholinergic nervous system.

#### 2.5.1. G-Protein Coupled Cholinergic Muscarinic Receptors

Muscarinic receptors responding to the neurotransmitter Ach have a widespread organ distribution and are involved in the control of numerous autonomic nervous system neurophysiological responses. This family of G-protein-coupled receptors consists of five members designated M1–M5 [194,195]. The gene family as a whole shows 26.3% overall amino acid identity, with the variation between the receptor subtypes being seen largely within the intracellular loops. The third intracellular loop is particularly variable, showing only 2.7% identity between receptor, compared with an average of 66% identity found in the conserved transmembrane domains. Classically these receptors are sub-divided into two broad groups based on their primary coupling efficiency to G-proteins. Hence, M2 and M4-muscarinic receptors are able to couple to the Gi/o-proteins and M1, M3 and M5-muscarinic receptors couple to Gq_/11_-proteins [195,196]. M3 type muscarinic receptors participate in contraction of smooth muscle, ileum, iris and bladder [197,198]. M2-muscarinic receptors [198] are also found in smooth muscle but play a minor role in the contractile response [196]. In the heart tissue however, their expression has a profound role in controlling cardiac myocyte contraction [196,197]. The muscarinic receptor family can couple with a wide range of diverse signaling pathways, some mediated by G-proteins and others that are G-protein-independent [199,200]. The binding pocket of the muscarinic receptor family is highly conserved. The discovery of snake toxins with relative selectivity for muscarinic Ach receptors should avail itself in studies of biochemical, pharmacological and functional properties of these different subtypes of muscarinic receptors [1].

##### Dendroaspis Toxin

The muscarinic toxins (MT) were originally purified from the venoms of the African mamba snakes [201] and were identified by binding to the muscarinic Ach receptors (mAChR-m**_1-5_**), but not to the nicotinic Ach receptors. The snake venom contains more than 12 different toxins affecting ligand binding. The MT1-MT7 were isolated from *Dendroaspis angusticeps* while MTα, MTβ and MTγ were isolated from the *Dendroaspis polylepis* [193] (Figure 1 *portal* 5). The MT2 ligand, which is the most abundant toxin present in the green mamba venom, is found at a concentration of 10 mg/g venom and accounts for approximately 50% of the total amount of venom toxins. Unlike MT2, the MT7 ligand was found at a 100-fold lower concentration [193]. The small three-finger proteins containing 65–66 residues and a molecular weight varying between 7–7.5 kDa with a high sequence identity were characterized [192]. The highly specific sequence signatures of these toxins include (i) eight cysteine residues involved in four disulfide bonds; (ii) an LTCV N-terminal motif; (iii) a TDKCNX C-terminal motif; (iv) a CP (D/A) GQN (L/V) CFK sequence in the region connecting loops I and II; and (v), a GC(A/V)ATCP sequence in region connecting loops II and III [193,202]. Despite their primary structure similarity, MTs interact differentially with the muscarinic receptor subtypes. For example, the MT7 is most potent and selective for mAChR-m**_1_** with a Ki of 14 pM. MTα is potent but not selective in binding to mAChR-m**_2-5_** with a Ki in the range of 2–40 nM while MT3 is potent and very selective for mAChR-m**_4_** with a Ki of 2 nM [193]. The LD_50_ for the MTs has yet to be determined, but no lethality (no death) was observed upon i.v. injection of 50 mg/kg MTβ analog to mouse [203]. Early pharmacological studies of MT toxins indicated, as expected, cholinomimetic activity such as evoked Ach release from brain corpus striatum, memory facilitation in an inhibitory avoidance learning task upon injection into the hippocampus and reducing twitch response of rabbit vas deferens preparations upon electrical stimulation [204]. Since mamba venoms contain also potassium channel antagonist, as well as other toxins, synergistic effects seem to be of great importance in the venom action and therefore it is likely that the muscarinic toxins are more potent when injected with other venom toxins. Given this remarkable diversity of MT toxins in snake venoms, should enable the generation of a library of GPCR compounds to be used in pharmaceutical development of drugs with higher selectivity for muscarinic receptor subtypes [192]. Today by virtue of their relative selectivity, MT toxins are used as pharmacological tools for identification, localization and activity of muscarinic receptors in the brain frontal cortex as well as in striatal neurons projecting into the globus pallidus in order to understand their role in the cognition, memory and movement [201].

#### 2.5.2. GTP Binding Proteins (G-Protein)

G-proteins are heterotrimeric proteins composed of a nucleotide-binding α-subunit (Gα) and a dimer consisting of a, β and γ subunits (Gβγ) [205]. In their inactive form, Gα-subunits are bound to guanosine diphosphate (GDP) and are tightly associated with Gβγ. Interactions between GPCRs and Gs (the stimulatory G protein that activates adenylyl cyclase) form the foundation of the ternary complex model of GPCR activation, in general. In the ternary complex consisting of an agonist, a receptor and a G protein, the affinity of the receptor for the agonist is enhanced and the specificity of the G protein for guanine nucleotides changes favors GTP over GDP. Agonist binding to the receptor promotes interactions with the GDP-bound Gsαβγ heterotrimer, leading to the exchange of GDP for GTP and the functional dissociation of G protein into Gα-GTP and Gβγ subunits. These separate subunits can modulate the activity of different cellular effectors such as ion channels, kinases or other enzymes amplifying receptor occupancy signal. The intrinsic GTPase activity of Gα leads to hydrolysis of GTP to GDP and the re-association of Gα-GDP and Gβγ subunits with termination of signaling. The active state of a GPCR can be defined as the stable conformation that couples and stabilizes the nucleotide-free G protein. The α-subunit (Gα) of the heterotrimeric G proteins mediates signal transduction in a variety of cell signaling pathways and are targeted by cholera toxin (Figure 1 *portal* 5). These α-subunits can be divided into four families: Gαs, Gαi/Gαo, Gαq/Gα_11_, and Gα_12_/Gα_13_. Each family comprises various members that often show specific expression patterns. The βγ-complex of mammalian G proteins is assembled from a repertoire of five G protein β-subunits and twelve γ-subunits [206]. Most GPCRs are able to activate more than one G protein subtype. Therefore, the activation of a GPCR usually results in the activation or inhibition of several signal transduction cascades via G protein α-subunits as well as through the free βγ-complex. The Gαs family is stimulating the adenylate cyclase (AC), the effector component in the transduction of GPCRs signal upon ligand occupancy. A typical example discussed below is *cholera toxin* [207], which targets these Gαs proteins. On the other hand, the Gαi/Gαo family inhibits AC upon coupling due to stimulation of inhibitory GPCRs. Another example discussed below is the *pertussis toxin*, which targets with great specificity the Gαi/Gαo family [208] (Figure 1 *portal* 5).

##### 2.5.2.1. Cholera Toxin (CT)

CT (Figure 1 *portal* 5) is a protein produced by the bacterium *Vibrio cholera*e and targets G-protein coupled receptors. CT is responsible for the excessive, watery diarrhea, characteristic to cholera infection [209,210,211]. The molecular weight of CT is 84 kDa and is present as an oligomeric complex of six subunits of two types: one copy of the A subunit containing the enzymatic activity, and five copies of the B subunit comprising the receptor binding structure [210,211,212,213,214]. Each of the five B subunits weights 12 kDa and forms a five membered ring organization [212,213,214]. The central cylindrical pore (outer diameter of 6–7 nm) is aligned by five amphipathic α-helices that are intimately involved in the pentamer stabilization [210]. The A subunit has two distinct domains, A1 and A2. Activation of the A subunits requires proteolytic cleavage of the loop that links A1 to A2 and a reduction of the disulfide bridge (Cys187–Cys199) that holds the two domains together [210,212,213]. The A1 domain is folded into a triangular shaped structure and is responsible for the enzymatic ADP-ribosylation activity, whereas the α-helical A2 domain is responsible for tethering the A1 domain to the pentameric B subunit. In doing so, the A2 domain protrudes right through the central pore of the B pentameric complex [210,212,213,214,215]. This syringe-like organization ensures the internalization of CTA subunit upon toxin binding to the cell [212]. CTA1 catalyzes the ADP-ribosylation reaction on GSα. This modification inhibits the GTPase activity of GSα and favors GDP>GTP exchange. The consequence is a permanent activation of GSα [216]. CTA1 contains a NAD^+^ glycohydrolase (NADase) and an ADP ribosyl transferase enzymatic activity. It has also 4 groups of substrates: simple compounds which contain guanidine group proteins with an arginine residue (like GSα), auto ADP-ribosylation of CTA1 and only hydrolysis of NAD [216,217]. The reaction itself is stereospecific when β-NAD serves as substrate. Kinetic experiments show that the reaction proceeds an SN2-like mechanism [217]. The CT B subunit (CTB) is a lectin that binds to apical membrane receptors, who in turn triggers toxin uptake and delivery of the A subunit in the target cell cytosol [209,214]. The CTB subunit has a high affinity for binding to GM1 ganglioside, a glycosphingolipid found in the outer leaflet of the plasma membranes of virtually all cell types, including enterocytes and lymphocytes [210,212,213,214]. Each B subunit has a GM1 binding pocket [209,210,214]. The B subunits interact mainly with the terminal galactose and to a lesser extent, with the sialic acid and N-acetyl-galactosamine moieties of GM1. Upon receptor binding, a flexible loop comprising amino acids 51–58 becomes more ordered due to hydrogen-bond interactions with the GM1 as well as water molecules that occupy the binding site. These interactions are believed to stabilize the toxin-GM1 complex [214]. The affinity of interaction between the CT holotoxin and GM1 is higher than that of the B subunit alone [213,214,215]. CTB binds to other gangliosides, yet at lower affinities and without known pathological significance [214]. Upon binding to GM1, CT is internalized in endocytic vesicles and then rapidly sorted to the trans-Golgi network (TGN) to enter a retrograde trafficking pathway to the endoplasmic reticulum [210,214]. This pathway depends on: (i) its association with detergent-insoluble glycosphingolipid-rich membrane microdomains (ii) the transport being initiated in early endosomes and (iii) cholesterol presence [214]. Once transported to the trans-golgi network, CT is thought to enter a retrograde trafficking pathway to the ER, best characterized as COPI-coated vesicles [213,214]. Upon delivery of CT to the ER, it has been suggested that the A1 domain is released from the A2-B pentamer complex and ER-resident chaperones and enzymes are then thought to be involved in facilitating translocation of an unfolded A1 domain into the cell cytosol. Then it exhibits ADP-ribosyltransferase and NAD-glycohydrolase activity and irreversibly ADP-ribosylates G- protein targets [210,213,214,215]. As G proteins are involved in the regulation of a large number of essential metabolic pathways in eukaryotic cells, this has an impact on numerous metabolic processes [214]. The main intracellular target that is directly associated with the induction of fluid secretion is Gs, a G protein involved in the activation of the stimulatory subunit of the adenylate cyclase complex [210,211,213]. This irreversible ADP- ribosylation inhibits the GTPase activity of the α subunit of Gs and results in constitutive activation of the adenylate cyclase complex and elevation of intracellular cAMP levels and its consequences [213,214,215]. One such event is the activation of the intestinal sodium pump via a cAMP-dependent protein kinase which precipitates a massive influx of sodium and water into the gut lumen [210,211,213,215]. Recent findings suggest that the cholera toxin B subunits do not simply bind GM1 gangliosides, but also possess independent signaling properties [214]. LD_50_ of cholera toxin in mice upon i.p. injection is 1.6 mg/kg [218]. Studies in humans have shown that the ingestion of 25 µg of CT induces heavy diarrhea [212]. CT has been widely used to understand the molecular mechanism underlying signal transduction mediated by Gs [212,214] and also as a neuronal tracer to follow up retrograde axonal transport. The CTA1 subunit in particular, has been used as a tool to investigate Gs protein-coupled receptors mode of action [219], as well as signaling cascades stimulated by cAMP. The high affinity binding of CTB subunit to the ganglioside GM1 has been instrumental in drug delivery via liposomes to target drugs and proteins to mucosal tissues, while recombinant mutants of CT were generated for vaccines production [219].

##### 2.5.2.2. Pertussis Toxin (PTX)

PTX (Figure 1 *portal* 5) is an exotoxin produced by the gram negative bacterium *Bordetella pertussis*, which targets specifically Gi protein-coupled receptors. The holotoxin comprises of 952 amino acid residues, has a molecular weight of 105 kDa and is forming six dissimilar subunits [220] that are non-covalently linked and arranged in S1–S5 stoichiometry [221]. PTX is a member of the AB5 toxin family [222]. The B moiety is a pentameric protein complex composed of two dimmers S2–S4 and S3–S4, joined together by S5 [223], which is responsible for specific binding to eukaryotic target cells receptors [224] and for toxin delivery to the target cell [222]. The A moiety, composed of 234 or 235 amino acids [224], forms a single peptide subunit S1, which expresses an ADP ribosyl-transferase enzymatic activity [221,223]. In the absence of eukaryotic protein substrates, the A subunit catalyzes the hydrolysis of NAD to ADP-ribose and nicotinamide. The A subunit contains two cysteines at positions 41 and 201 in a disulfide bond [224], an essential form for the catalytic activity [220]. The crystal structure of PTX has been determined at 2.9 Å resolution [220] and has revealed that the B pentamer does not contain a central pore [222]. Instead the S2/S3/S4/S4/S5 subunits are arranged as a triangular platform on top of which the S1 subunit rests like the tip of a pyramid [221]. A protomer is transported across the plasma membrane to its target sites within the cell as the result of the binding of PTX to the cell surface via the B oligomer moiety [225]. Through the S2 and S3 subunits, the B domain of PTX binds to cell membrane glycoproteins with a branched mannose core and an N-acetyl glucosamine attached [225]. The binding site on each of these two subunits is a sugar chain containing a terminal sialic acid, that position on the “shoulders” of the pentamer, near the catalytic subunit [222]. The S1 subunit is thus translocated inside the target cell where it interacts with G proteins and blocks their activity [225] by binding NAD and transferring the ADP-ribose to a cysteine 351 residue [222]. The latter is present in the XCGLX motif of the C-terminus of the α subunit of many GTP-binding proteins, such as Gi, Gt, Go and Ggust [225]. The covalent modification of the cysteine 351 by ribosylation induces a conformational change involving the C-terminus. This change opens the active site cleft, allowing entry of substrates, which decouple the G protein α subunit from its receptor [220]. Site-directed mutations confirmed that the C terminal domain of S1 is involved in G-protein binding, while the N-terminal domain contain the NAD^+^-binding site and the residues involved in catalysis. S1 subunit also catalyzes the cleavage of NAD^+^ into ADP-ribose and nicotinamide [221]. Binding of the PTX-B oligomer to a plasma membrane sialylated glycoconjugate leads to Gi protein inactivation and cell toxicity, whereas binding to a non-sialylated molecule, leads to a rapid signaling response. The question as to whether PTX binds to different sites for early signaling events and G-protein inactivation or binds to the same receptor leading to both stimulatory and inhibitory biological effects remains to be determined [223]. Most eukaryotic cells contain PTX receptors and acceptor substrates [221] and therefore respond to a variety of hormones or neurotransmitters which cause constitutive activation of adenylate cyclase, accumulation of cAMP, and lead ultimately to cell toxicity [225]. For example, following stimulation with PTX, pancreatic cells enhance insulin secretion, adipocytes induce fatty acid and glycerol release, macrophage migration is inhibited and Chinese hamster ovary cells show induced morphological changes [221]. In vivo administration of PTX induces lymphocytosis, a hallmark of systemic pertussis in children. It also causes hyperinsulinemia and hypoglycemia, modifications in vascular permeability and histamine sensitization [221]. Interaction of the B-oligomer with receptors on certain eukaryotic cells can also mediate biological effects that are independent of the catalytic activity of S1, including proliferation of T lymphocytes, agglutination of erythrocytes and adherence of bacteria to host cells [220]. PTX is lethal at 0.025 mg/kg in mice [226], but it induces toxic effects at a very low concentration. PTX has been instrumental in the discovery of G-proteins and the characterization of signal transduction events in eukaryotic cells. It is a useful pharmacological tool to determine the expression of Gi/Go/Gt proteins and whether a putative receptor is coupled to a cellular effector by the above mentioned G- protein [223,227]. PTX also plays an important role in the development of protective immunity to whooping cough. The progress in biotechnology and molecular biology allowed the creation of genetically detoxified PTX by producing several S1 mutants in E.coli. The PTX mutants carrying the substitutions at positions Lys9 and Gly129 show the most favorable knockout of their enzymatic and toxic activity [225]. This detoxified PTX is now the prime element of the highly immunogenic and efficacious acellular pertussis vaccine in infants [225].

#### 2.5.3. House-Keeping Enzymes

A large number of proteins are required for physiological activities in all cells throughout the organism. These proteins are sometimes called housekeeping proteins, indicating that their expression is crucial for the maintenance of basic functionality of all cells and therefore, ubiquitously expressed in every cell type of each tissue in the organism. A transcriptomics analysis of the housekeeping proteome [228] of samples representing all major human organs and tissues in the body identified 7367 protein-coding genes detected in all analyzed tissues (the human protein atlas) including different receptors, G proteins and their effectors such as adenylate cyclase, MAPKs and phospholipases.

##### 2.5.3.1. Adenylate cyclase

The adenylate cyclase (AC) (EC 4.6.1.1), is one of the most common house-keeping enzymes which plays key regulatory roles in the eukaryotic cell. It has been classified into six distinct families all catalyzing the same reaction but representing unrelated gene families with no known sequence or structural homology [229]. The best known AC class is class III which occurs widely in eukaryotes and fulfills important roles in signal transduction. AC catalyzes the conversion of adenosine triphosphate (ATP) to 3′, 5′-cyclic AMP (cAMP) and pyrophosphate. Mg^2+^ ions are generally required and appear to be important co-factors. The cAMP produced by AC is a regulatory second messenger signal, via specific cAMP-activated kinases (PKA). The latter are a major group of protein kinases transducing the activation of GPCRs by ligands to phosphorylate many intracellular substrates [230]. Several bacterial toxins, including *Anthrax toxin* [231] cause dysregulation of this essential house-keeping enzyme and its signaling cascade as detailed below.

###### Anthrax Toxin

Anthrax (Figure 1 *portal* 5) is an infection caused by pathogenic strains of *Bacillus anthracis*, which secret a three- component toxic complex consisting of protective antigen (PA), edema factor (EF) and lethal factor (LF) proteins, which potently and efficiently target cellular components of adaptive immunity [232], resulting in bacterial immune suppression [233]. While LF and EF are enzymes that act on cytosolic substrates, PA is a multifunctional protein that binds to receptors and orchestrates the assembly and internalization of the complexes, and their delivery to the endosome [234]. PA is a 83 kDa protein containing four domains. Domain 1 (residues 1–258) has the RKKR sequence, where the proteolytic activation of PA occurs after it binds to either of two cell-surface receptors, anthrax toxin receptor (ATR)/tumor endothelial marker 8 (TEM8); and/or capillary morphogenesis protein 2 (CGM2) [235,236] which interact with LDL receptor-related protein 6 (LRP6), essential for the following step of endocytosis. Proteolytic cleavage by furin at the cell surface, removes a 20 kDa fragment (PA20) from the N-terminus of PA [237] leading to PA_63_ [238]. Cleavage is required for assembly of seven or eight cleaved PA_63_ into heptameric or octameric pre-pore complexes, with high binding affinities for LF and EF (Kd~1 nM). Clustering the toxins into specific cholesterol and glycosphingolipid-rich micro-domains of the plasma membrane promotes clathrin-dependent endocytosis. The pH reduction in the endosomes triggers (PA_63_) 7 pre-pores to undergo an acidic pH-dependent conformational rearrangement to form an ion-conducting, cation-selective, transmembrane pore, allowing bound LF and EF to translocate into the cytosol [234,235,239]. The EF is a 89 kDa protein containing 767 amino acids with adenylate cyclase activity that requires activation by calmodulin (CaM) [240,241]. Structures of the EF-CaM complex indicate that EF locks the N-terminal domain of CaM into a closed conformation regardless of its Ca^2+^-loading state [242]. EF being almost 1000 fold more potent then endogenous AC, strongly increases the cAMP to pathological levels which results in impairment of all intracellular signaling pathways responsible for AC activation. By this mechanism, the endogenous AC activity stimulated by GPCR is minimal, compared to the cAMP induced by EF in the intoxicated cells. EF enter the cytosol from late endosomes, remains associated with this cellular compartment and shows a perinuclear localization, generating intra cellular cAMP concentration gradients from the cell center to the periphery [243]. We may consider this molecular mechanism as comparable to over expression of AC in those cells. The end result will be persistent activation of substrates regulated by cAMP-PKA induced phosphorylation. An additional molecular mechanism contributing to anthrax toxicity is induced by LF. LF is a 90 kDa protein which contains 776 amino acids and is a Zn^+2^-dependent metalloprotease, consisting of 4 domains. The first domain is involved in PA binding, while the fourth domain contains the catalytic center of the enzyme that binds to the mitogen-activated protein kinase kinases (MAPKK) on the NH_2_-terminus. MAPKKs are activated by tyrosine kinase receptors and play important roles in cell survival, growth, proliferation and differentiation. Binding of LF results in a shut-down of the several MAPK kinases such as MEK1, MEK2, MEK3, MEK4, MEK6, and MEK7 [244] the end result of which may be cell cycle arrest and/or cell death [240]. This LF mechanism is inactivating the major tyrosine kinase receptors induced- Ras/MEK/ERK pathway. In summary, the presence of both LF and EF in the cell contribute to toxicity by dysregulation of the GPCR systems (Figure 1 *portal* 5) and tyrosine kinase receptors (Figure 1 *portal* 7) signaling. Commonly, the LD_50_ of the whole anthrax toxin (PA/LF/EF) was characterized by spore number per mice and therefore, cannot be precisely translated into mg/kg mice. Since PA alone is non-toxic but required for internalization of the toxic factor LF and EF, in general, mice toxicity is evaluated by combination of PA + LF or PA + EF [245]. The LD_50_ toxicity values estimated for the pairs PA:LF or PA:EF at a ratio of 1:1 upon i.v. injection to mice, were 0.25–2.4 mg /kg [246] or 1.25–3.75 mg/kg [247], respectively. It is predicted that lethality of the whole anthrax toxin is achieved by additive pathological activity of these multi-functional PA/LF/EF proteins. The MAPK pathway is activated under normal physiological conditions to regulate signaling emerging from both tyrosine kinase and GPCR. Therefore, studies indicating that treatment with lethal doses of the anthrax toxin cause tumor regression [248], inhibit growth [240] and inhibit angiogenesis [249], propose that LF can be utilized in tumor research and drug development. Moreover, LF modified anthrax fusion proteins have been used to deliver HIV antigens [250]. PA efficiently transports antibody mimetics into mammalian cells [251], can be used for detection of anthrax toxins using a nano aptasensor approach [252] and is an useful mucosal adjuvant [253]. Regarding EF, there are no studies indicating its use as pharmacological tool; however, we can propose that EF in conjunction with PA or by iontophoretic micro-injection can be introduced into cells to generate a strong cAMP/PKA activation signal. Definitely, all three anthrax toxins protein components can be used for development of new therapeutic and/or diagnostic agents [254].

##### 2.5.3.2. PI-specific phospholipase C (PLC)

Another important house-keeping enzyme is the phosphatidylinositol (PI) specific-PLC (EC 3.1.4.11) which catalyzes the hydrolysis of phosphatidylinositol 4,5-bisphosphate (PIP_2_) to IP_3_ and DAG both of which are essential second messengers [255]. Metabolic degradation of PI plays a vital role in a wide variety of cellular functions [256]. IP_3_ diffuses through the cytosol to specific intracellular receptors to promote release of Ca^2+^ ions from the endoplasmic reticulum and cause an influx of extracellular Ca^2+^. DAG is essential for activation of some isoforms of protein kinase C. but is also a source for AA. Thus, PI degradation by PLC involves production of multiple second messengers which regulate Ca^2+^-dependent processes, protein kinase C-mediated protein phosphorylation reactions and AA-associated pathways [257]. PI-PLC are toxins secreted by many gram positive bacteria [258]. PI-PLC secreted by *Staphylococcus aureus* is a typical toxin [259] interfering with the physiological IP_3_ signaling pathway (Figure 1 *portal* 5).

###### *Staphylococcus aureus* (*S. aureus*) PI-PLC Toxin

*S. aureus* PI-PLC toxin (Figure 1 *portal* 5) secreted by strains of the bacterial *Staphylococcus* is a protein of 34.1 kDa. The enzyme catalyzes the cleavage of membrane lipid PI and membrane protein anchors containing glycan- phosphatidylinositol (GPI) [260]. The PI-PLC enzyme consists of a single domain folded as a (α/β) (8)-barrel (TIM barrel), is calcium-independent and its catalytic domain involves two histidine residues [258,261]. PI-PLC removal of the protective GPI-anchored proteins on the exterior surface of mammalian cells accelerate cell death and DAG translocation across the bilayer [262] and can interfere with cellular signaling processes inducing different pathological effects [263]. *S. aureus* PI-PLC cleaves its substrate via an intramolecular phosphotransferase reaction on the PI moiety to form a cyclic inositol phosphate molecule and DAG. Subsequent hydrolysis of the cyclic phosphodiester produces inositol 1-phosphate if PI is the substrate [261,264]. The *S. aureus* PI-PLC activity towards PI is unique and its optimal activity is at acidic pH [260]. The availability of *S. aureus* PI-PLC toxin may provide an efficient biochemical tool to characterize the role of GPCR /PI-PLC signaling in different cellular functions. 

### 2.6. Toxins Acting on Small GTP-Binding Proteins

The small GTP binding proteins are a family of cytoplasmic GDP/GTP-binding proteins with GTPase activity (Rho, Ras, Rac, Raf), that serve as biomolecular switches inside the cell to regulate a variety of cellular processes [265]. The activation/deactivation cycle of these small GTP-binding proteins is tightly regulated by guanine nucleotide exchange factors (GEFs), GTPase-activating proteins (GAPs), and guanine nucleotide dissociation inhibitors which catalyze the exchange of GDP by GTP and thereby activate these small regulatory proteins. Small GTP-binding proteins interact with a variety of effector proteins and promote downstream signaling events. They carry a C-terminal hypervariable region (HVR), which is the target of post-translational modification. Lipidation by farnesyl or geranyl cysteinyl thioethers of the HVR anchor the small GTP binding proteins to membranes and regulate their subcellular localization, including domains in the plasma membrane [266]. *Clostridium botulinum C3 exoenzyme* is a typical toxin that causes the addition of one or more ADP-ribose moieties to Rho A, B and C proteins [267,268] leading to its toxic effects (Figure 1-*portal* 6) [269,270]. In addition, *Pseudomonas aeruginosa type III secretory* Exoenzyme-S ADP- ribosylate Rac but also Ras, inhibiting signaling through Ras [271]. Therefore, modulation of small GTP-binding proteins by these microbial toxins may significantly inhibit a variety of plasma membrane receptors signals involving small GTP-binding proteins, resulting with pathologies and cytotoxic effects.

#### Botulinum C3 exoenzyme Toxin

The C3 enzyme (Figure 1 *portal* 6) is an exozyme which ADP-ribosylates small GTP-binding proteins such as Rho and has been isolated from *Clostridium botulinum* [267]. It is produced as a single polypeptide of 26 kDa with an N-terminal signal peptide of 40 amino acid residues cleaved during intracellular trafficking. C3 possesses an NAD- glycohydrolase activity generating ADP-ribose and nicotinamide from NAD [272]. The monomeric form of C3 is active and folds into a compact three dimensional structure containing α-helix and β-sheet conformations. A central cleft in the NAD-binding pocket is formed and packed as β sheet core and an α -helical segment [273]. The C3 core catalytic domain consists of two perpendicular antiparallel β-sheets that form a cleft at their interface. A conserved sequence (residues 174-176, the ‘STS motif’) is located at the end of the C3 structure where it plays both an important catalytic and structural role [273]. The central core of the C3 resembles the catalytic domains of similar ADP-ribosylating toxins including diphtheria toxin [273]. The C3 enzyme can modify all Rho isoforms at asparagine-41 site [274] leading to biologically inactive (GDP-bound) Rho [275,276], resulting in dramatic changes of the actin cytoskeleton [277]. The details of binding /or uptake the C3 enzyme to cells are unknown [273]. A nonspecific pinocytotic mechanism at high C3 concentration or after prolonged incubation has been proposed [278]. Brief exposure to low pH is sufficient to mediate direct translocation of C3 across the cell membrane. In macrophages C3 uptake occurs via receptor-mediated endocytosis [276]. Morphological changes such as rounding of the cells and destruction of stress fibers were noticed in Vero cells [279]. While there are approximately 180 small GTP-binding proteins, only C3 is currently used to study Rho A–C functional consequences on signal transduction processes [280,281]. In order to use this toxin as a biochemical tool, it has been introduced into cells by microinjection [282], or by using a cell-permeable chimeric structure between C3 and the binding domain of diphtheria toxin (C3–DT) [283]. Experiments of knock down of Rho A–using siRNA show a massive Rho B expression and activation. This undesirable side effect was due to a suppression of the Rho B promoter by Rho A suggesting that the two are mutually related [284]. Strong Rho B expression is also observed upon treatment of cells with C3, but Rho B turned completely inactive by C3. Thus, presently, application of C3 is the major experimental approach to effectively inhibit Rho A without concomitant Rho B activation [267]. 

### 2.7. Toxins Affecting Tyrosine Kinase Receptors (RTKs)

The RTKs are a family of cell-surface receptors such as epidermal growth factor receptor (EGFR), which transduce signals derived from hormones, cytokines and growth factors. They regulate general metabolic processes and cell functions such as proliferation, differentiation, cycle, survival, and motility [285]. All RTKs display an extracellular ligand binding domain, a single transmembrane helix, a cytoplasmic region containing the protein tyrosine kinase activity. Agonist binding to the extracellular domain causes dimerization and oligomerization of RTKs. That leads to auto-phosphorylation of the tyrosine kinase catalytic domain in a trans-orientation, serving as a site of assembly of protein docked complexes and stimulation of multiple signal transduction pathways, including MAPK, PLC-γ and PI_3_-kinase [286]. To the best of our knowledge no toxins have yet been discovered which bind directly and modulate RTKs. However, pore forming toxins such as *Staphylococcus α-toxins*, by increasing intracellular Ca^2+^, activate the Ca^2+^-depended protein kinases and decrease the affinity of RTKs, contributing to cytotoxicity (Figure 1-*portal* 7) [287].

#### *Staphylococcus aureus* α-Toxin

Detailed characteristics of the *S. aureus* α-toxin (Figure 1 *portal* 7) have been presented in Section 2.1.1.2. Studies in PC12 cells cultures have demonstrated that the toxin reduces the affinity of the EGFR to the epidermal growth factor (EGF) [287]. Treatment of cells with Na^+^ orthovanadate, a phosphatase inhibitor, before exposure to the α-toxin significantly reduced its effect on EGFR, suggesting that specific protein tyrosine phosphatases may shift the equilibrium towards dephosphorylation of EGFR [288]. An alternative explanation is that plasma membrane phospholipids perturbation after pore formation activates/translocates specific phosphatase(s) that cause dephosphorylation of EGFR resulting in decreased affinity. In addition to phosphatase translocation, activation of PLA_2_ by *S. aureus* α-toxin resulting in high intracellular Ca^2+^ and increase in eicosanoid content has been shown [289,290]. Since the α-toxin has an effect on the phosphorylation status of EGFR at low non-toxic level, the decrease in the receptor affinity to the ligand has been used in pre-clinical research of EGFR signaling. Moreover, as EGF receptor signaling is responsible for cancer progression, this toxin can be applied in experimental cancer therapy research as a specific inhibitor of EGF signaling and cell proliferation [291].

### 2.8. Toxins Acting on Integrins Receptors Affecting Cell Adhesion

The integrins comprise a family of receptors responsible for eukaryotic cell adhesion to the extracellular matrix (ECM) proteins, therefore playing an important physiological role in the maintenance of the tissue organization. These proteins are connected to intracellular proteins that couple to diverse signaling molecules recruited to sites of focal adhesion and are also linked to the actin cytoskeleton. These heterodimeric receptors are composed of non-covalently linked α and β glycoprotein subunits, containing a long extracellular domain binding to the ECM, a transmembrane domain, and a short cytoplasmic domain that associates with the actin cytoskeleton and adaptor proteins. Currently 18 distinct α and 8 β subunits of the integrin receptor family have been identified and cloned in mammals, which can combine in a restricted manner to form at least 24 heterodimeric assemblies. Each of the α–β heterodimers exhibits a distinct ligand-binding profile and is responsible for a distinct cellular function [292]. Two families of toxins collectively named disintegrins [293] and C-type lectins (CTL) [294] were isolated and characterized from snake venoms [295]. Disintegrins block integrins with relative high selectivity affecting cell adhesion, proliferation, migration as well as survival [296]. Below a detailed account is presented.

#### 2.8.1. Disintegrins

The disintegrins (Figure 1 *portal* 8) constitute a family of small (40–100 amino acids), cysteine-rich polypeptides that have been isolated from the North African sand viper, *Cerastes cerastes cerastes*, from *Vipera obtusa* and *Vipera palestinae* and other snake venoms. The disintegrins are classified into five different groups according to length (short, medium and long) and number of disulfide bonds (from 4 to 8) of the polypeptides [297]. The fourth subfamily of disintegrins contain the disintegrin-like domains derived from PIII-SVMPs, modular proteins containing an N-terminal disintegrin-like domain of about 100 amino acids including 16 cysteine residues involved in the formation of eight disulfide bonds, and a C-terminal 110–120-residue cysteine-rich domain cross-linked by six disulfides [298]. Unlike the short, medium and long disintegrins and PIII disintegrins, which are single-chain molecules, the fifth group is composed of homo- and heterodimers [295]. In many disintegrins the active motif is the tripeptide “RGD”, a motif responsible for affinity and selectivity towards integrins such as α_IIb_β_3_, α_v_β_3_ and α_5_β_1_. Obtustatin and Viperistatin, members of the disintegrin protein family contained the “KTS” binding motif and are very selective for collagen receptors α_1_β_1_ integrin [299]. Dimeric disintegrins exhibit the largest sequence diversity in their integrin binding motifs. EC3, a heterodimeric disintegrin containing an “MLD” motif in its B-subunit is a selective and potent antagonist of the binding of α_4_β_1_ and α_4_β_7_ integrins to immobilized VCAM-1 and MAdCAM-1, respectively. It is also a weak inhibitor of α_5_β_1_ and α_IIb_β_3_ integrins and does not inhibit α_v_β_3_ integrin [300]. EMF-10, another heterodimeric disintegrin is an extremely potent and selective inhibitor of integrin α_5_β_1_ binding to fibronectin and partially inhibiting the adhesion of cells expressing integrins α_IIb_β_3_, α_v_β_3_ and α_4_β_1_ to their appropriate ligands [301]. Selective recognition of α_5_β_1_ by EMF-10 is associated with the “MGD” sequence, a motif located in the active loop of the B-subunit. The presence of a “WGD” motif in CC8, a heterodimeric disintegrin from the North African sand viper, venom has been reported to be responsible for the relative selective inhibitory effect on α_IIb_β_3_, α_v_β_3_ and α_5_β_1_ integrins [302].

#### 2.8.2. C-Type Lectin-Like (CTL) Toxins

CTL toxins (Figure 1 *portal* 8) show a high sequence homology (15%–40%) to the carbohydrate recognition domain of the C-type lectins [303,304]. Structurally, CTLs are heterodimers composed of homologous α and β subunits with molecular weights of 14–15 and 13–14 kDa, respectively. Both covalent and non-covalent polymerization of these heterodimers are possible, giving yield to αβ, (αβ)_2_ and (αβ)_4_ structures [303]. In contrast to CTLs, classic C-type lectins form exclusively homodimers linked by an inter-chain disulfide bridge; these can also polymerize to oligomers [305]. Classical C-type lectins aredivided into seven subgroups, according to their structural characteristics. CTLs play a role in adhesion, endocytosis and pathogen neutralization [306]. Some of the main targets of CTLs are membrane receptors, coagulation factors, and proteins essential to hemostasis. Adhesion receptors of platelets, such as the von Willebrand factor (vWF)-binding GPIb-complex, the collagen-binding GPVI and integrin α2β1, and the fibrinogen receptor integrin αIIbβ3, are essential in platelet activation and aggregation. Accordingly, some CTLs are antithrombotic and anticoagulative while others may activate coagulation factors [307]. CTLs have been used in elucidating some mechanisms of the coagulation cascade and integrins-induced signaling pathways. EMS16, Rhodocetin, and VP12 are important CTLs which inhibit collagen-dependent cell adhesion and migration. They represent important tools to investigate tumor metastasis and angiogenesis [294]. Disintegrins and CTLs from snake venom are relatively not toxic (LD_50_ in mice upon i.v. injection is higher than 10 mg/kg) and therefore constitute useful tools to studies of integrin receptors role and signaling in cell adhesion and migration [296] and as lead compounds towards development of new drugs [308].

### 2.9. Toxins Acting on the Nucleic Acids

Transcription is the first step of gene expression, whereby a particular gene (DNA) is translated into mRNA which in turn, acts as a template for protein’s synthesis. Histones are protein components binding DNA of nuclear chromatin and playing a role in gene regulation [309]. Histone deacetylases (EC 3.5.1.98, HDAC) consist of a class of enzymes that remove acetyl groups from the ε-N-acetyl lysine amino acid on histones, allowing the protein to wrap DNA more tightly and therefore amenable to acetylation and de-acetylation [310]. Mycotoxins, such as *Aflatoxin B1*, are secondary metabolites of fungi that exert toxic effects on animals and humans by forming DNA adducts [311] and Trichostatin A by blocking histone deacetylase activity [312]. In both cases disturbance in gene transcription and carcinogenesis and teratogenesis may ensue as outlined below (Figure 1 *portal* 9). Another mechanism by which toxins may affect DNA is by endonuclease activity such as DNAse1 which cleaves DNA preferentially at phosphodiester linkages adjacent to a pyrimidine nucleotide, yielding 5′-phosphate-terminated polynucleotides with a free hydroxyl group on position 3', producing a tetra-nucleotides. This novel group of toxins isolated from gram negative bacteria is the cytolethal distending toxins (CDT) which encode a multi-subunit toxin [313,314]. However, the neurotoxicity of CDT was not yet investigated and its LD_50_ not yet reported, and therefore, this group of toxins will not be further addressed.

#### 2.9.1. DNA-Targeted Mycotoxins

Mycotoxins (Figure 1 *portal* 9) are “toxic secondary metabolites” produced by several distinct fungi-including *Aspergillus, Fusarium, Penicillium, Stachybotrys*, and *Myrothecium* species. Fungi can produce from one to several mycotoxins. Mycotoxins have no specific molecular feature in common and therefore cannot be classified within a defined chemical category. Basically they comprise of peptide derivatives (i.e., cyclochoritme), ergot (gliotoxin, sporidismine), quinone (lotuskirin, rogulosin), peron (aflatoxin, citrinin, kojic acid, sterigmatocystin), terpine derivatives (i.e., fusarinone, satratoxin, trichothecines, vomitoxin), nonadrid (rubratoxin), alkaloid (i.e., lisergic acid, slaframin), xanthine (sterigmatocystin), lacton (i.e., patulin, penicillic acid, rubratoxin, zearalenone) and coumarin group including the aflatoxin as well as other mycotoxins (i.e., ochratoxin, sterigmatocystin) [315]. A most notorious mycotoxin is Aflatoxin AFB_1_, which causes chromosomal aberrations, micronuclei generation, sister chromatid exchanges, DNA synthesis impairment, chromosomal strand breaks, and formation of adducts in rodent and human cells. Covalent binding to DNA is a characteristic property of the aflatoxins which contain an unsaturated terminal furan epoxide-forming ring. Studies on the reaction of synthetic AFB_1_–8,9-epoxide with DNA in vitro strongly indicates that adduct formation proceeds by a pre-covalent intercalation complex between double-stranded DNA and the highly electrophilic, unstable AFB_1_–exo-8,9-epoxide isomer. The *exo* isomer of AFB_1_–8,9-epoxide appears to be the only product of AFB_1_ involved in reaction with DNA and reacts with the *N*^7^ atom of guanine via an SN_2_ reaction from an intercalated state [316,317]. Covalent binding of AFB_1_ to adenosine [318] and cytosine [319] in DNA in vitro has also been reported. The predominant AFB_1_–DNA adduct was identified as AFB_1_–*N*^7^-Gua, which is derived from the covalent bond formation between C8 of AFB_1_–8,9-epoxides and the *N*^7^ of guanine bases in DNA. The formation of AFB_1_–*N*^7^-Gua is linear over the low-dose range in all species examined, and liver, a primary target organ, has the highest level of the adduct. Formation of initial AFB_1_–*N*^7^-Gua adduct has been correlated with the incidence of hepatic tumor in trout and rats. Initial AFB_1_–*N*^7^-Gua adduct can convert to a ring-opened AFB_1_–FAPY derivative. Accumulation of AFB_1_–FAPY adduct is time-dependent, non-catalytic, and may have potential biological impact (i.e., DNA defects) because of its apparent persistence in DNA [320]. The role of AFB_1_–DNA damage in the mechanism for AFB_1_ carcinogenesis has been extensively studied. Replication of DNA containing AFB_1_–*N*^7^-Gua adduct-induced G→T mutations in vivo. Activation of the *ras* protooncogene has been found in AFB_1_-induced tumors in many animals including mouse, rat and fish. More strikingly, the relationship between aflatoxin exposure and development of tumorogenicity is further demonstrated by the studies on the *p53* tumor suppressor gene [321]. High frequency of *p53* mutations (G→T transversion at codon 249) was found to occur in populations exposed to high levels of dietary aflatoxin in China and South Africa [322,323]. Ingestion of mycotoxins causes a range of toxic responses, from acute toxicity to long-term or chronic health disorders [324]. Mycotoxins induce diverse and powerful biological effects which are carcinogenic, mutagenic, teratogenic, estrogenic, hemorrhagic, and they are toxic to the skin, to the immune system and to organs such as kidney, liver and brain. A single mycotoxin can cause more than one type of toxic effect. The LD_50_ of oral delivery of AFB_1_ in rats and rabbits is in the range of 0.4- 2.7 mg/kg [325] and in guinea pigs of 2 mg/kg [326]. Due to their carcinogenic potency, mycotoxins including aflatoxins, sterigmatocystin, ochratoxin, fumonisins, zearalenone, and some *penicillium* toxins, are used as pharmacological tools in experimental animal models for understanding tumorigenicity. Most of these carcinogenic mycotoxins are genotoxic agents with the exception of fumonisins. While repair of AFB_1_–DNA adducts in bacteria is relatively well understood, further research is needed to fully characterize mammalian repair of these adducts. *S. cerevisiae* is a robust model system to study DNA repair, and studies in yeast have been important in further understanding the mechanism of repair in eukaryotic cells. Since all studies have investigated repair of AFB_1_-induced DNA damage in either whole animals or intact cells, more mechanistic studies are needed to confirm that the presumed mutagenic adducts, AFB_1_-N^7^-Gua and AFB_1_–FAPY, actually are repaired specifically by the nucleotide excision repair (NER) pathway. Furthermore, the contributions of other pathways such as base excision repair (BER), and DNA mismatch repair (MMR), warrant further study in both mammals and bacteria [317]. Additional investigation of the mechanism by which AFB_1_ can modify repair activity is of significant interest, to improve our understanding of the mechanism of AFB_1_ carcinogenesis, and possible mechanisms of action of other carcinogens [317].

#### 2.9.2. Histone Deacetylases Toxins

Known as natural Histone Deacetylases (HDAC) inhibitors (Figure 1 *portal* 9), these toxins are of fungal or microbial origin. Trichostatin A (TSA), the first specific and most investigated HDAC inhibitor was isolated from the fermentation broth of *Streptomyces hygroscopius* [327]. TSA and synthetic hydroxamic acid inhibitors (e.g., suberoylanilide hydroxamic acid, SAHA) contain a cap phenyl group, an aliphatic chain, and a terminal hydroxamic acid functional group [312,328,329]. Co-crystallization of a bacterial HDAC homolog with TSA or SAHA demonstrated that the catalytic site of HDAC has a tubular pocket structure with a Zn^2+^ cation in its base. HDAC inhibitors mimic lysine groups of the natural substrate. Their interaction with the active site prevents the binding of histone tails, thereby blocking enzymatic deacetylation [330]. TSA coordinates the Zn^2+^ cations by the hydroxamic group. The aliphatic chain is inserted into the pocket and makes contacts with the hydrophobic groups lining the channel. The cap group interacts with residues at the rim of the pocket [330]. TSA has the optimal conformation to fit into the active site [330] and a high potency, (IC_50_ of 3.4 nM) in vitro [312]. However, only the natural, R-configuration of TSA can inhibit the enzyme effectively at nanomolar concentrations [331]. HDAC inhibitors cause accumulation of acetylated histones in both normal and tumor tissues. However, these compounds selectively induce the expression of only a small subset of genes that are involved in inhibition of cell cycle, activation of differentiation and induction of apoptosis [332]. One of the better studied pathways is the induction of the expression of CDKN1A gene, which encodes the cyclin-dependent kinase inhibitor p21. The latter inhibits cell cycle progression, causing cell cycle arrest at G1 phase [333]. In addition, in vivo indirect antitumor effects of these compounds include activation of the host immune response and inhibition of angiogenesis [333]. Histone acetylation and deacetylation are dynamic processes also in the adult brain. Accumulating data suggest that neurological activity, in conjunction with intracellular Ca^2+^, may regulate HATs and HDACs function [334]. Normal cells are usually less sensitive to the effects caused by HDAC inhibitors, compared to their transformed counterparts. However, under certain physiological conditions or during embryonic development, tissues, including the nervous system, may become sensitive to the effects of these agents. TSA are teratogenic in *Xenopus laevis*, presumably via HDAC inhibition [335]. Furthermore, TSA induces apoptosis in cultured neurons [336]. TSA administered i.p. to pregnant mice at a dose of 0.5–1 mg/kg at post-implantation stages (embryonic day 8 to embryonic day 10), is non-toxic for the mother during post-implantation stages (embryonic day 8 to embryonic day 10) and does not cause any obvious malformations [337]. Generally, HDAC inhibitors are well tolerated at the doses required to inhibit tumor growth, and their toxicities in both humans and animals are distinct from those associated with conventional chemotherapeutic drugs [332,333]. Phase I clinical studies with HDAC inhibitors in patients with various types of tumors have evaluated optimal dosage schedules required to achieve clinical outcomes while avoiding pronounced toxicity. Hydroxamic acid derivatives caused little or no toxicity in rodents at doses that markedly inhibited tumor growth, as assessed by weight loss and post mortem histological studies [332,333]. The first hydroxamic acid analog used in clinical trials, SAHA, was well-tolerated in humans [332]. A major concern for the long term is that HDAC inhibitors may have the potential for activation of oncogenes or epigenetically silenced genes [333]. HDAC inhibitors have been useful tools for isolation of HDAC enzymes and for studying the function of histone acetylation in cellular proliferation, differentiation and apoptosis [312,338,339]. HDAC inhibitors reduce growth of tumors and metastases in animal models [332,333,335]. Based on these results, several HDAC inhibitors are currently in Phase I and Phase II clinical trials, although the use of other compounds is limited due to their toxicity or poor bioavailability. The synthetic inhibitor SAHA causes tumor regression and induces symptomatic improvement in patients with either solid tumors or Hodgkin’s disease at well tolerated doses [332]. HDACs have been suggested as targets for the development of antiparasitic [340] and antiepileptogenic drugs [341] and for the treatment of Huntington’s disease [342]. Further development may provide more selective non-toxic HDAC inhibitors.

### 2.10. Toxins Acting on the Mitochondrial Respiratory System

Mitochondria are double membrane-bound organelles found in most eukaryotic cells where they provide most of the cell supply of adenosine triphosphate (ATP). The central set of reactions involved in ATP production is collectively known as the Krebs cycle [343]. In the mitochondrial electron transport chain, electrons move from an electron donor (NADH or QH_2_) to a terminal electron acceptor (O_2_), via a series of redox reactions. These reactions are coupled to the generation of a proton gradient across the mitochondrial inner membrane. There are three proton pumps: I, III, and IV. The resulting transmembrane proton gradient is used to generate ATP via ATP synthase. Electron transport inhibitors (ETS) act by binding different proteins on the electron transport chain, literally preventing electrons from being passed from one carrier to the next. They all act specifically, that is, each inhibitor binds a particular carrier or complex in the ETS. Irreversible inhibition results in a complete block of respiration along the NADH pathway, along the succinate pathway, or along the pathway that is common to both routes of electron entry [344].

#### Rotenone Toxin

The rotenone molecule (Figure 1 *portal* 10) is a ketonic toxic compound, currently used as a broad-spectrum pesticide. It occurs naturally in the seeds and stems of several plants, such as the jicama vine plant, and in the roots of several members of *Fabaceae* family. Rotenone crosses the blood brain barrier and plasma membrane by free diffusion due to its high hydrophobicity. Rotenone-induced degeneration of dopaminergic neurons has been noticed in CNS. Rotenone is an ETS which blocks the transfer of electrons from iron-sulfur centers in complex I to ubiquinone. It interferes with NADH during the generation of ATP. As a result, complex I is unable to transfer its electron to CoQ, creating a back-up log of electrons within the mitochondrial matrix [345]. The cellular O_2_ present is subsequently oxidized to a free radical, thus forming reactive oxygen species, which can damage DNA, lipids and other components of the mitochondria. The LD_50_ of rotenone after oral delivery, to rodents and humans is 350–1500 mg/kg [346] and 300–500 mg/kg [250], respectively. Rotenone is an important biochemical compound to study the mitochondrial respiratory system and oxidative stress insults in different diseases in particular Parkinson’s disease [347].

### 2.11. Toxins Acting on the Ribosome

The ribosome is a complex intracellular machinery that serves as the site for protein synthesis-translation of messenger RNA (mRNA) to proteins. The ribosomes comprise basically a small ribosomal subunit (40S) which reads RNA, and a larger subunit (60S), which enables amino acids to form the nascent polypeptide chain. The large subunit is composed of a 5S RNA (120 nucleotides), 28S RNA (4700 nucleotides), a 5.8S RNA (160 nucleotides) subunits and 46 different proteins [348]. On the ribosome template, elongation is the most rapid step in translation, using elongation factors (EF) which catalyze the formation of the first peptide bond to the formation of the last one in the peptide chain [349]. In this review inhibition of protein synthesis will be exemplified by diphtheria toxin which inhibits protein synthesis in the cell by inducing NAD^+^-dependent ADP ribosylation of EF-2 [350]. Notable, there are other toxins from plant origin, such as ricin, which deglycosylate the 28S ribosomal RNA and lead to the impaired ability of the ribosome to bind EF-2 [351]. 

#### Diphtheria Toxin (DT)

Diphtheria toxin (Figure 1 *portal* 11) is an exotoxin secreted by the aerobic gram-positive pathogen of bacillus *Corynebacterium diphtheria* [352]. It is a single polypeptide chain of 535 amino acid residues with a molecular weight of 58 kDa. It consists of three key domains: the amino-terminal C, or catalytic domain (residues 1–186); the intermediate T, or transmembrane, domain (residues 202–381); and the carboxyl-terminal R, or receptor-binding, domain (residues 391–535) [352]. These three structural domains are connected by disulphide bridges. The catalytic domain located in the C terminus is found in fragment A while the transmembrane (T) domain together with receptor-binding (R) domain are found in fragment B [353,354]. The B fragment is responsible for the entry into the cell via binding to a cell surface receptor accompanied by subsequent translocation of the toxic A fragment into the cytoplasm [352,355]. The native DT binds, via its R domain, to the heparin binding EGF protein precursor on the cell membrane, where it is cleaved by cell-surface furin or by a furin-like protease [356,357]. The di-chain protein that is still linked by a single disulfide bond between Cys186 and Cys 201 is internalized into clathrin coated pits and reaches the lumen of a developing endosome. This bond has to be intact in order for DT to deliver its active domain to the cytosol. In case this cysteine bridge is reduced before membrane translocation, the toxin is inactive [358]. DT’s toxic effect occurs by furin proteolytic cleavage of Cys186 and Cys201, followed by reduction of this inter chain disulfide bridge, resulting in di-chain protein containing A (21kDa) and B (37kDa) chains [357], and therefore, this toxin belongs to the AB bacterial toxins group. Upon endosome acidification (~5.3), the T domain undergoes a conformational change that leads to exposure of hydrophobic amino acid sequences that are inserted into the membrane, forming a channel through which the catalytic domain translocates and escapes from the endosome, probably with the aid of cytosolic factors [359,360,361]. The translocation of the C domain depends on the presence of cytoplasmic factors including β-COP and ATP. β-COP is a coatomer protein, which participates in the formation of trafficking vesicles from the early to the late endosomes [362]. In the cell cytoplasm, the catalytic domain exerts its toxic activity by transferring ADP-ribose from NAD to a modified histidine residue (diphthamide) at position 715 in the eukaryotic translation elongation factor (eEF2). This reaction forms a covalent bond between ADP-ribose and EF-2 which blocks the functional site which interacts with RNA in translation, and consequentially preventing all protein synthesis and causing programmed cell death [350,363,364]. Delivery of a single molecule of the catalytic domain into the cytosol is sufficient to cause cell death by inhibition of protein synthesis, indicative of the extreme high potency of this bacterial toxin [365]. The toxin has an estimated LD_50_ for humans of 0.1 µg/kg [366]. DT and its mutants can be used as pharmacological tools to study the contribution of eEF_2_ in protein synthesis. The major clinical use of DT is for preparation of antibody-toxin conjugates (immunotoxins) for cancer therapy. DT conjugated with interleukin-2 (IL-2) such as Denileukin and Diftitox (Ontak) have been produced to target hematologic malignancies such as adult T cell leukemia (ATL), chronic lymphocytic leukemia, Hodgkin’s and non-Hodgkin’s lymphomas, cutaneous T cell lymphoma (CTCL) and other leukemia and lymphomas [354]. ONTAK was approved by the FDA for treatment of advanced CTCL. Furthermore, this drug has been tested for treatment of other malignancies and non-malignant diseases like B-cell NHL [367], psoriasis [368], ovarian cancer, melanoma and Graft-versus-host disease (GVHD) [369]. Human GM-CSF was fused with DT and was successfully tested in phase 1 clinical trial in AML patients which were resistant to chemotherapy, and was submitted for phase 2 clinical trials [354]. Transferrin receptors (TfRs)- DT conjugate which lacks receptor binding activity [354] and Interleukin-3 (IL3)- DT conjugate are other examples of DT-immunotoxins used in clinical studies [370]. 

### 2.12. Toxins Acting on Exocytotic Vesicles

Exocytosis is the most common cellular mechanism for cellular secretion and consists of the fusion of an intracellular vesicle to the plasma membrane. In exocytosis, secretory vesicles carry their content across the cell membrane into the extracellular space. The exocytosis process is a chain of biochemical steps that involved vesicle docking, priming, fusion and release of the content [371]. It is spatially organized, such that opening of Ca^2+^ channels allows rapid translation of the entering Ca^2+^ signal into the fusion event [372]. A highly important exocytotic processes is associated with vesicular neurotransmitter release at the presynaptic site [373]. These unique membrane-bound vesicles contain not only neurotransmitters but also soluble proteins, lipids and ATP to be secreted into the synaptic cleft [374,375]. Several families of vesicle proteins have been characterized among which synaptophysin, synapsins, synaptobrevins andsynaptotagmins are most common [372]. The two toxins detailed below the *Botulinum* and *Tetanus* are both endowed with a metalloprotease activity which acts towards degradation of these unique proteins, after a long journey through the nerve cell, to ultimately lead to central paralysis and ultimate death [376]. 

#### 2.12.1. Botulinum Neurotoxin (BoNT)

The botulinum neurotoxins (BoNT) along with tetanus neurotoxin (TeNT) (Figure 1 *portal* 12) belong to a family of neurotoxins produced by different strains of the *Clostridium* bacteria [377,378]. *Clostridium* bacteria belong to six phylogenetically distinct groups and produce more than 40 different BoNT types [379]. BoNTs share a similar molecular weight and a common subunit structure; however, they are serologically different [377]. BoNTs are synthesized as single-chain polypeptides of ~150 kDa. The neurotoxin is activated following a specific proteolytic cleavage within a surface-exposed loop. The cleavage gives rise to a di-chain molecule composed of a 100 kDa heavy chain (HC) and a 50 kDa light chain (LC) that remain linked by a disulphide bond and by non-covalent interactions. Cleavage can be done by endogenous or exogenous proteases [380,381]. The HC sequence is poorly conserved among the neurotoxins, thus supporting the notion that different BoNTs bind different receptors [380,381]. The LC is a metallo- endopeptidase associated with a Zn^2+^ atom. Each BoNT has unique substrate and cleavage site despite identical Zn^2+^ -binding motif. The Zn^2+^ -binding motif (His-Glu-Xaa-Xaa-His) is located in a central region of the LC surface (216–244 region), inside a deep cleft, accessible by a channel. The Zn^2+^ atom is penta-coordinated by the imidazole rings of the two histidine and a water molecule bound to the glutamic acid of the motif [380,381]. The toxin enters the peripheral cholinergic terminus and through proteolytic degradation of specific vesicle proteins, inhibits the release of acetylcholine from synaptic vesicles [378]. Toxicity occurs through a multi-step process involving each one of the functional domains. BoNTs have developed a sophisticated strategy to passage the gastrointestinal tract and to be absorbed in the intestine of the host to finally attack neurons. A non-toxic non-hemagglutinin (NTNHA) forms a binary complex with BoNT to protect it from gastrointestinal degradation. This binary M-PTC is one component of the bi-modular 14-subunit ~760 kDa large progenitor toxin complex. The other component is the structurally and functionally independent dodecameric hemagglutinin (HA) complex which facilitates the absorption on the intestinal epithelium by glycan binding. Subsequent to its transcytosis the HA complex disrupts the tight junction of the intestinal barrier from the basolateral side by binding to E-cadherin. As a result, the toxin complex can enter the circulation by para-cellular routes in much higher doses [382]. Once in circulation, the dissociated BoNTs diffuse in the body to the presynaptic membrane of cholinergic nerve terminals, where it binds by its HC domain, and is then internalized inside intracellular vesicles. Internalization requires binding via polysialogangliosides to motor neuron presynaptic site at the neuromuscular junction. Subsequently, additional specific binding to luminal segments of synaptic vesicles proteins like SV2 and synaptotagmin leads to their uptake [382]. BoNT/B was shown to bind the synaptic vesicle protein synaptotagmin II, only in the presence of the ganglioside [380,383]. It was proposed that ganglioside-binding brings BoNT into close proximity with a protein receptor, facilitating toxin recognition and internalization [381,384,385]. After binding, BoNT enters the lumen of vesicular structures in a temperature and energy-dependent manner. At this stage, the toxin can no longer be neutralized by antisera [381]. BoNT HC domains are structurally homologous with low sequence identity, an important factor in determining the specificity of each toxin for its protein co-receptor [381]. The LC must leave the vesicle to exert toxic effects. It is proposed that there is a pH-dependent rearrangement of the toxin structure inside an acidic compartment in the cell that leads to greater hydrophobicity of the molecule that facilitates penetration of transmembrane regions in BoNT through the lipid bilayer forming an ion channel [381]. Thioredoxin reductase-thioredoxin protein disulfide-reducing system is present on the cholinergic synaptic vesicles and is responsible for the reduction of the interchain disulfide of botulinum neurotoxin serotypes A, C, and E. The BoNT metalloprotease activity is enabled upon disulfide bond reduction and causes neuroparalysis by cleaving the SNARE proteins [386]. BoNT LC cleaves SNARE proteins [“SNAP (Soluble NSF (N-ethylmaleimide-sensitivefusion) Attachment Protein) Receptor”] inhibiting exocytotic Ach release. Cleavage of the SNARE proteins is highly specific. No two serotypes cleave the same bond. The Zn^2+^ atom can be exchanged with another divalent transition metal ion forming active toxin while mutations in the Zn^2+^ binding motif inhibit catalytic activity [387]. BoNT interacts specifically with a nine-residue motif within the SNARE proteins, ‘SNARE secondary recognition (SSR) motif’ that is distinct from the cleavage site. Botulism toxicity is characterized by a flaccid paralysis starting in the head, neck, and upper extremities, followed by lower body paralysis and respiratory collapse [378]. BoNTs are the most potent toxins known, with LD_50_ estimated in a range of 0.1–1.0 ng toxin/kg upon i.p. injection in mice [29]. Unfortunately, there are discrepancies in the LD_50_ doses of different BoNTs serotypes in particular between research BoNTs and FDA approved clinical preparations with LD_50_ in the range of 0.1–5 ng/kg mice [388,389]. Brain toxicity upon i.c.v. injection to mice of BoNT serotypes A and B was characterized by LD_50_ in the range of 0.5–1.0 ng/kg [390]. The different BoNT serotypes exhibit differences in their toxic properties, depending on the cleavage site and how this disrupts SNARE complex formation [381,383]. Each of the BoNTs cleaves a different peptide bond in synaptobrevin II or SNAP-25 or syntaxin. Thus, BoNTs are well defined neurotoxin tools to identify the role the individual vesicular protein substrates, in different neuronal processes. Fine dissection of SNARE activities can be performed based on the specific peptide bond hydrolyzed by different BoNTs on the same protein [380]. An increase in number of docked vesicles in intoxicated synapses, suggest additional roles of SNAREs in exocytosis, and possibly involvement in vesicle re-uptake as well. The use of BoNTs is not limited to neuronal cells possessing BoNT receptors. Detailed protocols for the use of BoNTs on different cell type and organelles are available [380]. Most serotypes of BoNT hold therapeutic potential; however currently, only BoNT/A (BOTOX A^®^, Dysport^®^, Reloxin^®^, Azzalure^®^, Xeomin^®^, Bocoture^®^, PurTox^®^) and BoNT/B (MyoBloc^®^ /NeuroBloc^®^) are FDA-approved drugs in the clinic, with additional preparations in China and South Asia, to be used in a variety of cosmetic and neurological therapeutic applications [391]. Due to functional recovery, repeated doses are usually necessary to maintain therapeutic benefits. However large doses, given on repetitive occasions, can reduce the therapeutic effect most likely due to production of antibodies which neutralize the toxin. The majority of the immune response is generated toward the HC fragment, therefore future protein engineering of hybrid toxins could provide an avenue to prolong therapeutic efficacy. Patients that are immune to BoNT/A may benefit from injection of other serotypes [378].

#### 2.12.2. Tetanus Neurotoxin (TeNT)

TeNT (Figure 1 *portal* 12) produced by the *Clostridium tetani* bacteria under anaerobic conditions, is an extremely potent neurotoxin, only second to BoNT. The TeNT protein has a molecular weight of 150 kDa. It is translated from the gene as one protein which is subsequently cleaved into two parts: a 100 kDa heavy or B-chain and a 50 kDa light or A-chain. The chains are connected by a disulfide bond. The B-chain binds avidly to polysialogangliosides of neuronal membranes. It encompasses a translocation domain which aids the transfer of the protein across that membrane into the neuron [392,393,394,395] and neurotransmitter vesicle membrane [396]. Each domain of TeNT B-chain functions as a chaperone for the others and working in concert to achieve binding and membrane translocation [397]. Recently, nidogen, basal membrane entactin proteins enriched at the neuromuscular junction, were found to bind TeNT [398]. Therefore, both the polysialogangliosides [399], nidogens, and possibly other proteins [400] including the GPI anchored protein located in lipid microdomains are prerequisites for specific TeNT binding and internalization. Although ganglioside and protein receptor binding sites are in close proximity they function independently and do not require pre-formation of a ganglioside/protein receptor complex [401]. Once bound to these multiple receptors, the toxin is endocytosed into the nerve ending and begins a retrograde axonal transport trafficking through the axon, to the CNS spinal cord motor neurons [402]. TeNT is internalized in signaling endosomes shared with neurotrophins and their receptors, which are recruited to the fast axonal retrograde transport pathway [403]. In the CNS, TeNT once arriving at the first synaptic connection of the motor neuron, undergoes transcytosis, from the presynaptic motor neuron into the synapse space and thereafter into the postsynaptic inhibitory interneuron [403]. These trafficking stages are still not completely clear. Two pathways have been suggested: one that relies on vesicle recycling and the other one independent [404] of the synaptic vesicle 2 (SV2) systems. Once the vesicle is entrapped in the inhibitory interneuron, its translocation is temperature and pH dependent [405,406,407]. The translocated, TeNT undergoes a disulfide bond reduction, mainly by a “NADPH-thioredoxin reductase-thioredoxin redox system” and the light A- chain is free to cleave its substrate synaptobrevin on Gln76-Phe77 bond [408]. Cleavage of synaptobrevin [409] affect the stability of the SNARE core by restricting it from entering the low energy conformation which is the target for NSF binding [410]. Synaptobrevin is an integral V-SNARE necessary for vesicle fusion to membranes. The cleavages of synaptobrevin is the end target of TeNT and occur even at extremely low doses of TeNT, resulting with inhibition of GABA or glycine neurotransmitter exocytosis in the inhibitory interneurons. TeNT-induced paralysis and death are attributed to the blockade of these inhibitory neurotransmitters in the spinal cord causing disinhibition of the somatic, motor neurons, which release large amounts of Ach, causing the spastic contraction of the skeletal muscles. In the course of its action, TeNT also enhances PKC activity translocation and increases PIP_2_ hydrolysis in rat cerebral cortex preparations, indicative of additional mechanisms which can interfere with neurotransmitter release [411]. The LD_50_ of the toxin is approximately 0.05 μg/kg upon i.v. injection in mice [29]. TeNT may be usefully exploited for investigating the molecular basis of neuronal exocytosis [412], for its potential in inducing long-lasting plasticity changes in integrated brain functions such as epilepsy [413], as a drug delivery vector and for vaccine production [414,415].

### 2.13. Toxins Acting on Protein Kinases and Phosphatases

Protein kinases are group of enzymes that modify target proteins by addition of phosphate groups (phosphorylation) on either serine and/or threonine residues of cellular protein substrates, resulting in enzymatic modulation, cellular location, association with other proteins and functional changes. Analysis of the human genome suggests the presence of 518 protein kinases most of which regulate major cellular pathways that are essential in signal transduction [36]. The different kinases are classified according to the phosphate group-acceptor amino acid, into serine/threonine kinases (EC 2.7.11.1) and tyrosine kinases either receptor tyrosine kinases (EC 2.7.10.1) or non-receptor cytoplasmic tyrosine kinases (EC 2.7.10.2). The serine/threonine kinases can be further classified according to the second messenger activator such as PKA (cAMP), PKG (cGMP), PKC (Ca^2+^/DAG) and others [416,417]. The activity is regulated allosterically by a regulatory domain which keeps the enzyme inactive, until the second messenger is attached and the catalytic domain is activated. This is an important regulatory switch in signal transduction [416]. Phosphatases comprise a group of enzymes that remove the phosphate from the protein substrate, a reaction which is a reversal of phosphorylation. The most common phosphatases are protein phosphatases which remove the phosphate group from the phosphorylated serine/threonine (EC 3.1.3.16), or tyrosine (EC 3.1.3.48) amino acid residues of the substrate protein. Protein phosphorylation/dephosphorylation is a common posttranslational modification process. Several microbial, algae or fungal toxins which have high partition water/lipid coefficient due to their heterocyclic chemical structure, penetrate into cells and inhibit protein kinases or phosphatases leading to cell toxicity [418,419]. Several examples are detailed below.

#### 2.13.1. Staurosporine (St)

One of the most potent inhibitors of PKC is Staurosporine (St) (Figure 1 *portal* 13). St has been isolated from the culture broth of *Streptomyces staurospores* (*Streptomyces sp*. strain AM-2282), a soil microorganism well known for the production of the antibiotic Streptomycin. St (molecular weight of 466 Da) has a unique UV absorption [420] typical for an indole-carbazole chromophore with a sugar ring attached [421] and belongs to the K252 indole-carbazole family of protein kinase inhibitors. St also inhibits tyrosine kinases and other kinases due to its binding to the ATP site of the catalytic domain, a site highly conserved in most kinases [416,417]. Crystallographic analysis of the catalytic domain of PKA with St attached reveals binding to an adenosine pocket. This interaction provides an induced fit rearrangement of the enzyme and an open conformation. St uses both polar and non-polar interactions of the adenosine binding and generates new ones [422] and therefore, inhibits kinases by competition with ATP. The IC_50_ values for the serine/threonine kinases PKC, MLCK (myosin light chain kinases), PKA, PKG and CaMK (calmodulin dependent kinases) are 0.7–10 > 1.3 > 7–20 > 8–10 > 10–100 nM, respectively [417]. St is characterized by antimicrobial activity towards fungi and yeast and its injection in rodents caused a decrease in blood pressure [420]. In PC12 pheochromocytoma cells, keratinocytes and megakaryoblasts leukemia cells [423,424], St induces neurite outgrowth and filopodia formation. This process is attributed to changes in phosphorylation of cytoskeletal proteins, increase in tau protein levels, stabilization of microtubules [423,424] and changes in actin distribution [421,423]. The LD_50_ in rats and mice, upon i.p. and p.o. are 0.2 and 3–5 mg/kg, respectively. Upon cerebral administration (c.a.) in rats, the LD_50_ is reduced to 0.08 mg/kg and neurobehavioral symptoms such as excitability and changes in water demand are common. The minimal ratio between LD_50_ values by peripherally and central nervous system delivery is 2.5 indicating that St is the least toxic among the K252 family members [417]. That may be attributed to either binding to plasma proteins, rapid metabolism, or inability to cross the gastrointestinal tract and/or the blood brain barrier [417]. St cytotoxicity to different cells ranges from 0.1–1 μM which is 10–100 times higher than the concentration required for kinase inhibition [417]. St has been a major biochemical tool to unravel the role of PKC in signal transduction and apoptosis [417]. Because of its high toxicity [416,425], new analogs with potential use as drugs, have been developed. For example UCN-01 (7-hydroxy-staurosporine) a natural microbial alkaloid, was evaluated in phase II clinical trials for the treatment of some types of cancer. UCN-01 is a more selective inhibitor of PKC than St and inhibits cell cycle kinases and the P-glycoprotein responsible for the efflux of cytotoxic drugs [426]. Additional synthetic indole-carbazole analogs such as Gö6976 that do not bind to plasma proteins were developed [427]. 

#### 2.13.2. Okadaic acid (OA)

The marine microalgae *Halichondria okadai* and *Halichondria melandocia* secrete the 38-carbon polyether fatty acid called OA (Figure 1 *portal* 13). This toxin and its closed derivatives, dinophysistoxins and the OA-ester are responsible for the natural phenomena known as diarrheic shellfish poisoning (DSP) during red tides. OA potently and specifically inhibits the serine/threonine protein phosphatase 1C (PP1C) and protein phosphatase 2A (PP2A) [428], with an IC_50_ of 100 fold lower for PP2A inhibition (IC_50_ of 0.2 nM) than for PP1C inhibition [429]. Being very hydrophobic and therefore cell permeable [430], the differential inhibition of PP1C and PP2A activity in intact cells by OA has made this marine toxin a valuable biochemical tool for examining the role of phosphatases in a variety of cellular function. Modification of the OA carboxylic acid group on carbon C_1_ causes significant decreases in toxin phosphatase inhibitory activity [431,432] and therefore, OA with a methyl ester on C1 is completely ineffective as a phosphatase inhibitor. Methylation of the oxygen atoms associated with C2, 7, 24 and 27 also causes a significant decrease in toxin activity. These evidences suggest that the acidic moiety of OA is essential for toxicity. The LD_50_ of OA in mice, upon i.v. or i.p. injection, is 0.19 mg/kg [430]. In humans this toxin causes a self-limited diarrheal disease, probably due to increased phosphorylation of sodium-dependent transporters and aquaporin water channels, involved in water secretion by intestinal epithelial cells, similar to CTs symptoms [430]. In intact cells OA treatment produces a large increase in the phosphorylation of several proteins such as myosin light chain, elongation factor 2, acetyl CoA carboxylase, tyrosine hydroxylase, and modulates a variety of cellular functions such as smooth muscle contraction, fatty acid biosynthesis, protein synthesis and catecholamine synthesis and secretion [433]. Calcium influx through voltage-sensitive calcium channels (VSCC) may be increased by OA treatment since the channels are regulated by phosphorylation [434]. Recent data obtained in hippocampal neurons in culture have indicated that OA may increase the ionic influx through the ionotropic non-NMDA type excitatory amino acid (EAA) receptors, possibly by inhibiting the PP1 and PP2A activity [435]. OA micro-injection in rodent brain caused the appearance of “hyper phosphorylated” tau and neurodegeneration [436]. OA administered to rats by intragastric intubation caused intestinal damage, diarrhea and death, but no detectable effect on the liver. In contrast OA administrated intravenously had little effect on intestinal function, but caused a rapid dissolution of hepatic bile canalicular actin sheaths, congestion of blood in the liver, hypotension and death at high doses [437]. All together these data provide evidence for a variety of tissue selective toxic effects of OA in which inhibition of phosphatases caused dysregulation on protein kinase activity (increased phosphorylation) resulting in pathophysiological effects. For these reasons, OA has been a useful tool for studying the involvement of protein phosphatases in cellular processes. The effects of OA in cell-free extracts have led to the identification of several protein phosphatases’ substrates whose activities are controlled by phosphorylation. When tested on intact cells, OA mimicked the dramatic stimulation of glucose transport into adipocytes by insulin, suggesting that this process is mediated by phosphorylation of a protein on serine/threonine residues [433]. OA may also prove to be of value in identifying ion channels that are controlled by phosphorylation [430] as well as for the isolation and characterization of novel phosphatases isoenzymes. OA also serve as a lead compound in developing anticancer drugs, drugs for neurological disorders such as Parkinson disease, metabolic disorders, respiratory diseases and asthma [438].

## 3. Summary and Conclusions

Development of powerful arsenals to endow single prokaryotic as well as eukaryotic multicellular organisms of plant and animal kingdoms has been an essential feature throughout evolution, to enable survival of many species. This review attempts to address the question as to whether there are common denominators for the remarkable diversity in structure and function of biotoxic molecules (toxins) and how individual species have worked out in the phylogenetic tree molecular means to retain an advantage within their ecosystem. In this process, toxins have been shaped to selectively hit a key molecular target of the prey with essential physiological function. The diversity and molecular design of the seemingly different toxins discussed in this review represent a few examples for such tools, which enable survival of species. The first conclusion drawn from this elaborate and selected compendium of toxins, is that there are discrete *portals*, by which these biomolecules generated by the aggressor(s) accomplish the task of attacking other species at cellular and molecular levels. To this stage, 13 such *portals* have been identified and classified using a virtual cell model that may contain all these putative sites of interaction.

The 1st *portal* is the plasma membrane itself which may undergo permeabilization by reshuffling its microdomains via direct toxin penetration, to generate leakiness. Alternatively, amphipathic, pore-forming protein toxins bind with high affinity, insert and organize with a hydrophilic part lining a water-filled pore and a hydrophobic one, in contact with plasma membrane phospholipids, generating channels or holes. The deleterious imbalance of ion fluxes and water permeability accompanied by pore-induced leakage of cellular constituents overcome the immediate ability of the cell to regulate its intracellular milieu thus causing pathological and lethal effects. The 2nd *portal* is more complex because it involves several ions that are necessary to maintain the ionic equilibrium within the cell boundaries. This group, collectively termed ion channels, appears more elaborate, better conserved in function and tightly controlled. Indeed, ion channels are essential to both neural-excitable as well as to all cells of the living organisms. These channels transport ions at a very efficient and extremely fast rate. Neuronal, cardiac, skeletal or smooth muscle tissue maintain ionic gradient functions and membrane potentials using these channels, while in non-excitable tissue, e.g., cells from the immune system, skin or pancreas such as T cells, epithelium and β-cells, depend on the conductance of these channels to fulfill their individual functions. Many animal toxins serve as ligands of these ion channels, which are inactivated or kept open, resulting in very fast, deleterious imbalances, of ion fluxes in excitable tissues. Moreover, many of these ion channels are located in the synapse, the specialized structure enabling the activity and integration of nervous system and muscle function. Blocking these synapses with the toxin may result in paralysis of the organism. The 3rd and 4th *portals* are associated with Na^+^ and K^+^ ion homeostasis. While the 3rd *portal* covers energy-dependent ion pumps, the 4th *portal* of ligand-gated transmembrane ion channels (ionotropic receptors) in particular, requires the attacker to recognize the site of the physiological ligand i.e., glutamate or Ach, in order to establish contact. The latter *portal* is a typical *portal* of the excitable tissue, with which many of these toxins are engaged. Due to their high potency and selectivity, the toxins targeting these *portals* are powerful neurotoxins and cardiotoxins. The 5th *portal* comprising of G-protein coupled receptors is considered the most common system involved in signal transduction resulting from hormones, growth factors and different chemicals interactions. Ligand-binding to these metabotropic receptors directly leads to activation of intracellular signaling cascades. G proteins are involved in the regulation of a large number of essential metabolic pathways in eukaryotic cells. This *portal* includes members such as the muscarinic receptors that are activated by Ach neurotransmitter and have a widespread organ distribution and numerous autonomic nervous system neurophysiological responses. They include GTP binding proteins that modulate activities of different cellular effectors such as ion channels, kinases and other enzymes. Not of lesser importance within this group is the adenylate cyclase, a house-keeping enzymes, and certain enzymes of lipid metabolism which constitute essential features of the normal cellular physiology. Many microbial toxins are known to selectively and potently interfere with G-protein mediated signal transduction or with one of the steps downstream of this receptor system. The 6th *portal* encompasses a group of specific small GTP binding proteins which include Rho, Ras, Rac and Raf, cytoplasmic GDP/GTP-binding proteins with GTPase activity. Small GTP-binding proteins interact with a variety of effector proteins and promote downstream signaling cascades. The bacterial toxins which target and modify by specific enzymatic reactions small GTP binding proteins are most likely entering the cell by nonspecific pinocytotic mechanisms of which very little is known. The 7th *portal* consists of a family of cell-surface tyrosine kinase receptors (RTK) which transduce signals derived from hormones, cytokines and growth factors. They regulate general metabolic processes and functions such as proliferation, differentiation, cell cycle, motility, apoptosis and cell death. So far, toxins that directly bind and interfere with these receptors are unknown, but some pore forming toxins indirectly affecting these receptor’s tyrosine kinase phosphorylation signals, have been reported. The 8th *portal*, namely cell adhesion receptors, regulate cell-cell communication and migration. These receptors are tightly connected to intracellular proteins that couple to diverse signaling molecules recruited to sites of focal adhesion, and are also linked to the actin cytoskeleton. Snake venoms disintegrins and C-type lectin toxins, targeting with relative selectivity these integrin receptors, significantly affect the interaction between the cells and extracellular matrix of tissues, causing tissue disintegration. Although not a direct cell surface component, the 9th *portal* is a specific target for modulating the function of the transcriptosome. Toxins derived from either fungal or microbial origin, act through a subset of genes that are involved in cell cycle, differentiation and cell viability. Fungi-produced mycotoxins act as hepatotoxins, nephrotoxins, neurotoxins, and immunotoxins, according to the target organ. They can act as teratogens, mutagens, carcinogens, and allergens. Histone deacetylase (HDAC) inhibitors constitute a new class of cytostatic toxins with powerful anti-tumor activities through modulating acetylation/ deacetylation of histones and several transcription factors. The 10th *portal*, located also within the cell boundary, is associated with the regulation of the mitochondrial respiratory system, particularly the generation of proton gradients across the mitochondrial inner membrane. Here, plant toxins can interfere with the electron transport chain and cellular energy production. Passage of rotenone into the cell for example, is basically by free diffusion due to its hydrophobicity. In animal models, it crosses the blood brain barrier to compromise specifically dopaminergic neurons. The main targets however are mucous membranes i.e., gastrointestinal and respiratory organs’ epithelium. The 11th *portal* constitutes a route by which bacterial toxins compromise both translational and post-translational events. By perturbing protein synthesis, cell death cascades are initiated. The *portal* is in fact an EGF-like domain of the heparin-binding EGF-like growth factor (HB-EGF) precursor, present on the surface of cells, which binds the fragment B of diphtheria toxin. This is followed by entrapping the toxin inside an acidic endosome, where it is cleaved into its two fragments. Fragment A is subsequently released into the cytoplasm where it binds and change by a specific enzymatic reaction elongation factor-2 of the proteome machinery, to block protein synthesis. The 12th *portal* is a route by which mainly bacterial toxins interfere with vesicular release. The latter is a chain of reactions including docking and fusion of intracellular and extracellular membrane domains followed by vesicular content release, events which are controlled largely by Ca^2+^ ions. Exocytosis of small to large molecules is a phenomenon ubiquitous to most peripheral tissues. In the nervous system, exocytosis is a crucial process associated with vesicular neurotransmitter release, along with other soluble proteins, lipids and ATP. Passage of the BoNT and TeNT, the two most toxic compounds for humans, is attained by their binding to dual glycolipid and protein receptors, which are co-localized at nerve endings. They enter the nervous system at the neuromuscular junctions and/or migrate to the central nervous system by retrograde axonal transport and block exocytosis by cleaving different components of exocytosis machinery resulting with strong spastic (TeNT) or flaccid (BoNT) paralysis. The 13th and final *portal*, acts specifically on intracellular protein kinases and phosphatases to impair directly, second messenger-induced signaling cascades. Staurosporine, like other fungal toxins, can enter cells by passive diffusion across the plasma membrane similar to Okadaic acid, also a hydrophobic cell-permeable compound. By inhibiting protein kinases-phosphatases targets, regulation of the protein phosphorylation balance is impaired giving rise to a large variety of cellular pathologies.

Having defined these operationally clustered *portals*, we posed the question as to whether a conceptual bridge may exist between a group of seemingly different toxins acting at doses of ng/kg compared to mg/kg ranges, to understand the contribution and importance of their targeted *portal* cellular toxicity to the whole organism lethality. Evidently, from the scheme presented in Figure 1, it seems that following an initial interaction with the *portal* target, several basic second messenger pathways and protein kinases/phosphatases mechanisms are deregulated resulting in cytotoxicity. Even typical pore-forming toxins or ion channel modulators, although initially acting to disrupt the ionic equilibrium, they are also responsible for hyper-activation of second messenger-induced pathological reactions, due to influx and intracellular Ca^2+^ ions mobilization. 

Strikingly, an interesting correlation appears between the ng /kg doses required for the BoNT and TeNT to cause damage and the over a thousand fold dose required for example for the snake’s PLA_2_ toxins. This remarkable difference can be attributed to the extremely powerful *portal* through which the former toxins react as opposed to the later, and which involves blockade of neurotransmitters vesicular release that is critical for neuro-muscular junction function. Nevertheless, to increase lethality, unique PLA_2_ toxins have been generated throughout toxin evolution. Thus, two or more subunits, one recognizing a presynaptic target and a second with PLA_2_ catalytic activity have emerged, as β-Bungarotoxin, Crotoxin and Notexin for example. These toxins and their isoforms exhibit LD_50_ in mice in the range of 0.02–4 mg/kg upon i.p. or i.v. injections [212]. This higher potency of presynaptic PLA_2_ toxins may suggest that evolution is favoring functional diversification of toxins [439,440,441] targeting two or more *portals*. Other bacterial proteins such as pertussis and anthrax which deregulate *portal* 5, G-protein-coupled receptor systems, cause toxicity in the range of 0.1–1 μg /kg body weight, stressing the importance of this large group of receptors *portals* in maintenance of cellular functions. Since bacteria need the slow and sustained intoxication of the host cells to last for days to enable multiplication, they attack this *portal* to disconnect the metabolic machinery from G-protein-coupled receptor signaling. Untreated chronic infections with these bacteria, might eventually lead to these high amounts of toxin required to kill the host. Certain paralytic neurotoxins (i.e., Ω-conotoxins, Ω-agatoxin, etc.) targeting presynaptic Ca^2+^ channels subtypes in pain neuronal pathways are also characterized by toxicity in the range of 0.1–1 μg /kg body weight. The spider venom strategy for example is to paralyze the prey, enhance pain symptoms [442], but without causing rapid lethality. On the far end, toxins acting via ligand-gated ionotropic channel receptors, various PLA_2_ toxins derived from snake venoms and protein kinases/ phosphatases inhibitory toxins are characterized by intermediate toxicity in the range of 0.1–30 mg/kg body weight. One is tempted to suggest that in order to immobilize the prey at sub microgram range; venomous organisms secrete deadly cocktails of multiple toxins targeting different *portals*, to attain additive and synergistic interactions, thereby increasing lethality with a smaller amount of venom. Indeed, snakes have utilized the robust three-finger protein neurotoxin scaffold, targeting *portal* 4, to generate a group of toxins with wide variations in function involving subtle changes in the functional sites. Such a capability of adaptation offers the snake the flexibility to effectively capture its prey at their disposal [441]. Obviously, as the potency of the toxin increases, less amount of venom production is required to immobilize the prey thus providing a tool for small organisms to achieve an aggressive strategy. Finally there is an entire spectrum of toxins which include mycotoxins, disintegrins, C type lectins, HDAC toxins and animal pore forming toxins that all show LD_50_ values above 10 mg/per kg. That maybe in fact a miss representation for the actual potency as most of these viability tests are carried out in rodents as noticed before. When the latter are used, natural barriers such as the gastrointestinal wall and the blood brain barrier, are obstacles which can prevent the entry of undesirable substances of the type noted above. Cumulatively, these findings indicate the important role of signal transduction mechanisms to amplify toxin action leading to cell damage, irrespective of the *portal* it initially acts upon. Studies of these toxins in the context of the cellular targets have been most instrumental to develop toxinomimetic compounds which act either at the *portal* level or interfere with the process of bio-amplification, once the intracellular signaling pathways are stimulated. Drugs such as Botulinum toxin(s) blocking exocytosis [443], Ziconotide, an N-type calcium channel blocker [444], Integrilin, a selective platelet αIIIbβ3 integrin receptor antagonist [445], used in the clinic to treat a variety of pathologies, are all stemming from the recognition of the site of action in different cellular *portals*. Thus, in answer to the question posed above, we would like to propose that although there is a remarkable diversity between these *portals*, they are tightly linked to the amplification attained by the relatively limited cellular signaling transduction cascades. The recent development of venomics [446], toxins proteomics and genomics may facilitate rapid screenings for toxicity of molecules acting on this signal transduction machinery [447,448], based on safety as well as efficacy, thereby providing solid grounds for better drug discovery and translational medicine [449].
